# Transcriptional analysis of abdominal fat in genetically fat and lean chickens reveals adipokines, lipogenic genes and a link between hemostasis and leanness

**DOI:** 10.1186/1471-2164-14-557

**Published:** 2013-08-16

**Authors:** Christopher W Resnyk, Wilfrid Carré, Xiaofei Wang, Tom E Porter, Jean Simon, Elisabeth Le Bihan-Duval, Michael J Duclos, Sam E Aggrey, Larry A Cogburn

**Affiliations:** 1 Department of Animal and Food Sciences, University of Delaware, Newark, DE 19716, USA; 2 ABiMS, FR2424 CNRS-UPMC, Station Biologique, Roscoff 29680, France; 3 Department of Biological Sciences, Tennessee State University, Nashville, TN 37209, USA; 4 Department of Animal and Avian Sciences, University of Maryland, College Park, MD 20742, USA; 5 INRA, UR83 Recherches Avicoles, Nouzilly F-37380, France; 6 Department of Poultry Science, University of Georgia, Athens, GA 30602, USA

**Keywords:** Adipogenesis, Transcriptional regulators, Hemostatic genes, Lipogenesis, Adipokines, Retinoic acid signaling, Thyroid hormone action, Polygenic trait, Visceral obesity, Gene interaction networks, Canonical metabolic/regulatory pathways

## Abstract

**Background:**

This descriptive study of the abdominal fat transcriptome takes advantage of two experimental lines of meat-type chickens (*Gallus domesticus*), which were selected over seven generations for a large difference in abdominal (visceral) fatness. At the age of selection (9 wk), the fat line (FL) and lean line (LL) chickens exhibit a 2.5-fold difference in abdominal fat weight, while their feed intake and body weight are similar. These unique avian models were originally created to unravel genetic and endocrine regulation of adiposity and lipogenesis in meat-type chickens. The Del-Mar 14K Chicken Integrated Systems microarray was used for a time-course analysis of gene expression in abdominal fat of FL and LL chickens during juvenile development (1–11 weeks of age).

**Results:**

Microarray analysis of abdominal fat in FL and LL chickens revealed 131 differentially expressed (DE) genes (FDR≤0.05) as the main effect of genotype, 254 DE genes as an interaction of age and genotype and 3,195 DE genes (FDR≤0.01) as the main effect of age. The most notable discoveries in the abdominal fat transcriptome were higher expression of many genes involved in blood coagulation in the LL and up-regulation of numerous adipogenic and lipogenic genes in FL chickens. Many of these DE genes belong to pathways controlling the synthesis, metabolism and transport of lipids or endocrine signaling pathways activated by adipokines, retinoid and thyroid hormones.

**Conclusions:**

The present study provides a dynamic view of differential gene transcription in abdominal fat of chickens genetically selected for fatness (FL) or leanness (LL). Remarkably, the LL chickens over-express a large number of hemostatic genes that could be involved in proteolytic processing of adipokines and endocrine factors, which contribute to their higher lipolysis and export of stored lipids. Some of these changes are already present at 1 week of age before the divergence in fatness. In contrast, the FL chickens have enhanced expression of numerous lipogenic genes mainly after onset of divergence, presumably directed by multiple transcription factors. This transcriptional analysis shows that abdominal fat of the chicken serves a dual function as both an endocrine organ and an active metabolic tissue, which could play a more significant role in lipogenesis than previously thought.

## Background

The chicken was the first avian species and domestic animal selected for complete genome sequencing and assembly [1]. Subsequently, the chicken has emerged as a premier model in animal agriculture [2-4] and developmental biology [5]. Although now recognized as a model organism for biomedical research [6], the chicken has not been extensively used for the study of human diseases, especially metabolic disorders (i.e., insulin resistance, diabetes, obesity and metabolic syndrome). Several unique features of avian metabolism make the chicken an interesting model for understanding the interactions between genetic and endocrine factors that contribute to development of obesity and related metabolic disorders. In particular, chickens normally exhibit “hyperglycemia” [7,8], insulin resistance [8-11], hepatic *de novo* synthesis of lipids [12] and, like humans [13], abdominal (visceral) fatness is a polygenic trait [14-19]. Despite their relative insensitivity to insulin, acute immunoneutralization of insulin in the chicken provokes differential expression of more than a thousand genes in both liver and in skeletal muscle [20]. In contrast, only 69 genes were differentially expressed (DE) in abdominal fat of chickens following insulin immunoneutralization, albeit short-term fasting produced a much larger change (1780 DE genes) in transcription of abdominal fat genes [21]. This recent work also shows a rather large decrease in expression of lipogenic genes in abdominal fat of fasted chickens. A detailed examination of the insulin signaling cascade in adipose tissue of the chicken shows a distinct unresponsiveness to insulin [22]. Collectively, these observations support the chicken as a unique model for the study of the genetic and biological mechanisms controlling fatness or leanness.

Most mammalian models of obesity exploit single gene mutations or use high-energy, high-fat diets to induce obesity [23]. Our chicken models are two experimental lines of meat-type chickens that were divergently selected over seven generations for either high (FL) or low (LL) abdominal (visceral) fatness [24,25]. These chickens exhibit a 2.5-fold difference in abdominal fat weight at 9 weeks (wk) of age, albeit their body weight and feed intake are similar [26]. Furthermore, the FL chickens present hyperplasia and hypertrophy of adipocytes at an earlier age than do LL chickens [27,28].

Differential abundance of lipogenic genes in liver of the FL and LL chickens was determined earlier by differential mRNA display [29], quantitative RT-PCR [30,31] or targeted low-density array [32]. Our preliminary analysis of the liver transcriptome in the FL and LL chickens during juvenile development revealed 1,805 differentially expressed (DE) genes [3]. Quantitative trait loci (QTL) analyses of an FL x LL intercross identified a major QTL for abdominal fatness at the distal end of chromosome 5 (*GGA*5) [16,17,33]. Further, the expression quantitative trait loci (eQTL) analysis of *GGA*5, involving a three generation intercross of the FL x LL chickens, identified variations in expression of 660 hepatic genes that were correlated with abdominal fatness traits [19].

The present study has a dual purpose to explore the abdominal fat transcriptome of juvenile FL and LL chickens and to identify major gene networks controlling adiposity and lipogenesis in these divergently selected models. Using the Del-Mar 14K Chicken Integrated Systems cDNA microarray, we took transcriptional snapshots of gene expression in abdominal fat across two genotypes (four birds/genotype) and six ages during juvenile development (1–11 weeks of age). Interestingly, our time-course transcriptional analysis of abdominal fat revealed numerous DE genes (main effect of genotype or age × genotype interaction) that are involved in hemostasis (14 genes), adipokine signaling (8 genes), retinol metabolism (13 genes), and the synthesis (37 genes), oxidation (12 genes) and transport (12 genes) of lipids. The liver is widely considered as the major site of lipogenesis in chickens and other birds. However, the present transcriptional analysis of visceral adiposity has identified 37 lipogenic DE genes, including *FASN, SCD, SREBF1, SREBF2* and *THRSPA* that are expressed higher in FL chickens. The greater abundance of thrombogenic enzymes and related protease inhibitors in abdominal fat of the LL chickens suggests enhanced proteolytic processing of adipokines and other endocrine factors, with local and/or humoral actions, that could contribute to their leaner phenotype. Although abdominal fat is generally considered as a passive depot for lipids, the present descriptive study in FL and LL chickens supports our idea that it does contribute to lipid synthesis and serves as an endocrine organ, which liberates a host of adipokines and endocrine factors with intrinsic and/or extrinsic activity.

## Methods

### Animals and tissue collection

The birds were bred and raised at the Institut National de la Recherche Agronomique (INRA) UE1295 Pôle d’Expérimentation Avicole de Tours, F-37380 Nouzilly, France. At hatching, FL and LL cockerels were wing-banded and vaccinated against Marek’s disease virus. Birds were reared together in floor pens (4.4 × 3.9 m) and provided *ad libitum* access to water and conventional starter feed for three weeks [3,050 kcal of metabolizable energy (ME)/kg and 22% protein] and thereafter with a grower ration (3,025 kcal ME/kg and 17.9% protein). Chicks were held under continuous light (24 h or LL) for the first two days after hatching, followed by a 14 h light/10 h dark cycle (14L:10D) for the remainder of the experiment. Infrared gas heaters provided supplemental heat and ambient temperature was decreased weekly from 32 C at hatching until 22 C was reached at 3 wk of age. Eight birds from each genotype were randomly selected at six ages (1, 3, 5, 7, 9, and 11 wk), weighed, bled into heparinized syringes, and killed by cervical dislocation. Abdominal fat was quickly dissected and weighed; a sample was immediately snap frozen in liquid nitrogen and stored at −75 C until further processing. All animal procedures were performed under the strict supervision of a French government veterinarian and in accordance with protocols approved by the French Agricultural Agency, the Scientific Research Agency, and the Institutional Animal Care and Use Committees at INRA, Nouzilly, France. These procedures were also in compliance with the United States Department of Agriculture (USDA) guidelines on the use of agricultural animals in research and approved by the University of Delaware Agricultural Animal Care and Use Committee.

### Microarray analysis

Four birds per genotype and age were randomly selected from the total of eight birds sampled per genotype and age for microarray analysis of abdominal fat (Additional file 1). Total cellular RNA was extracted from abdominal fat using guanidine thiocyanate and CsCl gradient purification [34], followed by a separate step for DNase I treatment. The RNA concentration was determined with a NanoDrop ND-1000 spectrophotometer (NanoDrop Technologies; Wilmington, DE). RNA integrity was examined using an RNA 6000 Nano Assay kit and the Model 2100 Bioanalyzer (Agilent Technologies; Palo Alto, CA) to assess the quality of the RNA samples (RNA integrity number, RIN ≥ 9 was considered acceptable). Twenty μg of total RNA was indirectly labeled using SuperScript Plus Indirect cDNA Labeling System (Invitrogen, Carlsbad, CA). First strand cDNA synthesis was performed in a 30 μl final volume containing 1× first-strand buffer, 5 μg of anchored oligo(dT_20_), DTT, dNTP mix (including aminoallyl- and aminohexyl-modified nucleotides), 40 U of RNaseOUT and 800 U of SuperScript III reverse transcriptase with an incubation at 46 C for 3 h. The original RNA template was removed by NaOH hydrolysis, and followed by neutralization with HCl. The cDNA was purified using a low-elution volume spin cartridge (Invitrogen; Carlsbad, CA) and labeled with either Alexa Fluor® 555 or Alexa Fluor® 647 succinimidyl ester in the dark at room temperature for 2 h. After purification of labeled cDNA with a low-elution-volume spin cartridge, the efficiency of dye incorporation was determined using the Microarray Module on the NanoDrop ND-1000 spectrophotometer and the Base:Dye Ratio Calculator on the Invitrogen website [35].

Twenty-four Del-Mar 14K Chicken Integrated Systems microarrays (NCBI GEO Platform # GLP1731) were hybridized with 48 labeled samples using a balanced block design, where half of the birds from each genotype and age were labeled with Alexa Fluor® 647 (red dye) and the other half with Alexa Fluor® 555 (green dye; see Additional file 1 for details of the hybridization design). Hybridized slides were scanned using a GenePix 4000B scanner with GenePix Pro 4.1 software (Molecular Devices, Union City, CA) at wavelengths of 635 nm (Alexa Fluor® 647-labeling) and 532 nm (Alexa Fluor® 555-labeling) generating a combined TIFF image file for each slide. The laser power was set at 100% with the photomultiplier tube (PMT) setting being adjusted for each scan to produce a PMT count near unity. All slides were manually checked for quality and all spots with inadequacies in signal, background or morphology were eliminated from further analysis. The image analysis results were merged with Excel files in GenePix Report (GPR) format, which contains clone identification, spot location on slide, and most current gene name/function (based on BLASTX/BLASTN score).

The microarray GPR files were analyzed using the linear models for statistical analysis of microarray data (LIMMA, version 3.4.5) software [36] package in R (version 2.11.1) [37]. Median intensities for each dye were Loess normalized (without background subtraction) within array and between array (“Aquantile” method) to correct for dye and slide biases. A two-way analysis of variation (ANOVA) was used on Loess normalized intensity values from this factorial design experiment to determine the main effect of genotype (G), main effect of age (A), and the interaction of age and genotype (A × G). The Benjamini-Hochberg procedure [38] was used to control the experiment-wise false discovery rate (FDR) from multiple testing procedures.

### Quantitative RT-PCR analysis

Several DE and prior candidate genes were selected for verification of expression by quantitative RT-PCR (qRT-PCR) analysis. First-strand cDNA synthesis was performed by incubation of a 13 μl reaction volume (containing 1 μg of total DNase-treated RNA, 1 μl of 100 μM oligo dT_20_, 1 μl of 10 mM dNTP mix, and water to 13 μl total volume) for 5 min at 70 C and then placed on ice for 2 min. A master mix containing 5 μl of 5× first-strand synthesis buffer, 1 μl of 0.1 M dithiothreitol (DTT), 1 μl of RNaseOUT, and 200 U of SuperScript® III reverse transcriptase (Invitrogen, Carlsbad, CA) was added to the RNA in a final reaction volume of 20 μl. The cDNA was diluted to achieve a concentration of 50 ng/μl. Primers were designed for qRT-PCR using Primer Express® v2.0 software (Applied Biosystems, Foster City, CA). Detailed information for each primer pair including gene name, gene symbol, primer sequences (forward and reverse), GenBank accession number and amplicon size are provided in Additional file 2.

The qRT-PCR assay was performed in an ABI Prism Sequence Detection System 7900HT, using Power SYBR® green PCR master mix (Applied Biosystems, Foster City, CA) and 400 nM of each primer (forward and reverse; Sigma-Aldrich, St. Louis, MO) in duplicate wells. Disassociation curves of each sample were analyzed to validate specific amplification and verify absence of primer dimers. PCR products were analyzed using agarose gel electrophoresis to compare approximate product size to expected amplicon size. The Ct for each sample was normalized to the corresponding sample geometric mean of three housekeeping genes [protein kinase, AMP-activated, beta 2 non-catalytic subunit (*PRKAB2*), protein kinase, AMP-activated, gamma 1 non-catalytic subunit (*PRKAG1*), and serpin peptidase inhibitor, clade E (nexin, plasminogen activator inhibitor type 1), member 2 (*PAI-2* or *SERPINE2*)]. These housekeeping genes were selected using the RefFinder website [39] as the most stably expressed genes (i.e., genes with the lowest M-value) in the experiment. The 2^-(∆∆Ct)^ formula was used to calculate relative transcript abundance [40]. The statistical analysis was performed using a general linear model procedure in SAS v9.3. The data (log2 transformed normalized expression values) was analyzed using a two-factor analysis of variance to determine significant effects of genotype (G), age (A), and the interaction of age x genotype (A × G). Pearson’s correlation coefficient (r) was used to compare log2 FL/LL expression ratios between the microarray and qRT-PCR analyses of select genes.

## Results

### Phenotypic measurements

Body weight (BW, kg), abdominal fat weight (g), and relative abdominal fat content (percent of body weight, %BW) in juvenile FL and LL chickens are presented in Table 1. The BW of FL and LL cockerels was similar for all ages between 1 and 11 wk. The absolute and relative abdominal fat weights of the FL chickens were 2.5-fold higher (*P*≤0.01) than those of the LL at all ages between 3 and 11 wk of age.

**Table 1:** Phenotypic measurements from juvenile FL and LL cockerels

	**Age (wk)**					
	**1**	**3**	**5**	**7**	**9**	**11**
***Body weight (kg)***						
FL	0.115	0.544	1.297	1.983	2.693	3.222
LL	0.123	0.551	1.204	1.964	2.787	3.281
***Abdominal fat (g)***						
FL	0.5	13*	38*	88*	124*	150*
LL	0.4	5 *	15*	31*	54*	59*
**FL/LL ratio**	**1.2**	**2.6**	**2.5**	**2.8**	**2.3**	**2.5**
***Abdominal fat (%BW)***						
FL	0.4	2.3*	2.9*	4.4*	4.6*	4.6*
LL	0.3	1.0*	1.2*	1.6*	1.9*	1.8*
**FL/LL ratio**	**1.3**	**2.3**	**2.4**	**2.8**	**2.4**	**2.6**

Values represent the least square means (LSMEANS) of eight birds/genotype and age with a common standard error (not shown). Significance (denoted by *) between FL and LL was determined at *P*≤0.05 using Fisher’s least significance difference (LSD) test. Rows in boldface type present the FL/LL ratio of abdominal fat weight (g) and abdominal fat as a percent of body weight (%BW).

### Abdominal fat gene expression

**Figure 1:**
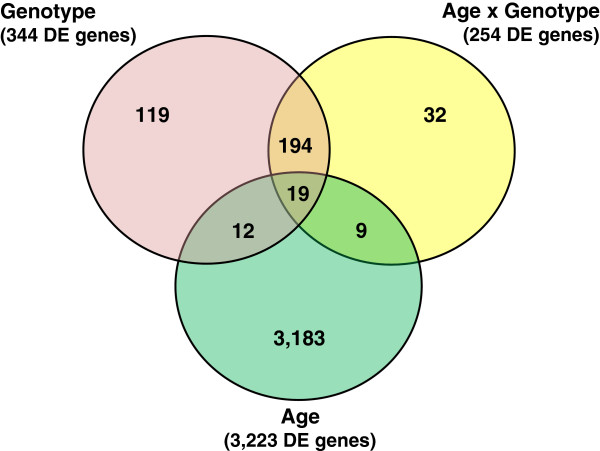
**Venn diagram showing unique and shared genes among main effect of age (A) or genotype (G), and their interaction (A × G).** This diagram shows the number of differentially expressed (DE) genes that are common across contrasts and those that are unique to G (*P*≤0.05), A (*P*≤0.001), or the A × G interaction (*P*≤0.05).

Differentially expressed (DE) genes were defined as those having a significant false discovery rate (FDR)-adjusted *P*-value. The significance level was set at *P*≤0.05 for genotype (G) and the age (A) × G interaction; or *P*≤0.001 for A. The statistical analysis of this factorial design (2 genotypes × 6 ages) experiment provided DE gene sets for the main effects of G and A, or the interaction of A × G. The main effect of G was determined by comparing gene expression values of each genotype (FL vs. LL) averaged across the six juvenile ages (1–11 wk). Likewise, the main effect of age (A) was determined by comparing gene expression values of each age averaged across both genotypes. To distinguish differences between ages, five single-degree-of-freedom contrasts were made by comparing the average of each subsequent age (averaged across both genotypes) against the 1 wk average (1 vs. 3 wk, 1 vs. 5 wk, 1 vs. 7 wk, 1 vs. 9 wk and 1 vs. 11 wk). The Venn diagram (Figure 1) shows the overall number of DE genes for G (344 genes), A (3,223 genes) and the interaction of A × G (254 genes) and the intersection of these DE gene sets. The number of unique genes are indicated for G (119 DE genes), A (3,183 DE genes) and the A × G interaction (32 DE genes). There were 213 DE genes in common between G and the A × G interaction. Thirty-one DE genes were shared between A and G, whereas 28 DE genes were in common between A and the A × G interaction. Overall, 19 DE genes were found in common among all three effects.

All DE genes involved in the higher order A × G interaction were removed from the main effects of A and G lists. Therefore, the total number of DE genes presented in the A × G interaction list (254 DE genes) in Additional file 3 reflects the 32 unique genes for the A × G interaction plus those genes shared with the main effects of A (19 DE genes) and G (194 DE genes). The number of DE genes presented in the main effect of A (3,195 DE genes) and A (131 DE genes) lists represent the total number of DE genes for that effect minus those genes that belong to the A × G interaction (Additional file 3).

### Ingenuity® Pathway Analysis (IPA®) of DE gene sets

Significant genes (cDNA clone IDs) from the microarray analysis were annotated using the GeneBase tool on our website [41], which provides protein IDs (from GenBank or Swiss-Prot databases) of microarray cDNA probes derived from BLASTX analysis. Lists of DE genes containing the protein ID and log2 ratio for each gene were then submitted to the Ingenuity® Knowledge Base [42] for functional annotation and mapping to canonical metabolic and regulatory pathways. “Analysis ready” genes were mapped by IPA for the genotype (100 DE genes), age (2,301 DE genes), and age x genotype interaction (212 DE genes) lists. The IPA® Upstream Regulator Analysis was used to identify transcription factor (TF) interaction networks, predicted activation or inhibition of TF, and their direct targets from DE gene sets.

A summary of the IPA “Diseases and Disorders” category under “Biological Functions” is presented in Table 2. The subcategories of major interest were “Developmental Disorder” (33 genes), “Hereditary Disorder” (71 genes), “Inflammatory Disease” (7 genes, out of which 6 were up regulated in LL chickens), “Metabolic Disease” (41 genes), and “Organismal Injury and Abnormalities” (31 genes). A group of 33 genes were classified as “inborn error of metabolism” in three of the above subcategories (Developmental Disorder, Hereditary Disorder, and Metabolic Disease) [see Additional file 4].

**Table 2:** Top biological functions of DE genes in abdominal fat of juvenile FL and LL chickens*

**Diseases and disorders**	***P*-value**	**# Genes**	
Developmental disorder	2.76E-07	33	
Hereditary disorder	3.01E-06	71	
Inflammatory disease	7.14E-06	11	
Organismal injury and abnormalities	4.51E-05	31	
Metabolic disease	4.77E-05	41	
**Molecular and cellular functions**			
Lipid metabolism	6.06E-05	46	
Small Molecule biochemistry	6.06E-05	43	
**Physiological system development and function**			
Hematological system development and function	1.87E-05	34	
Organ morphology	2.56E-05	7	
Renal system development/function	2.56E-05	10	
Embryonic development	1.06E-04	23	
Cardiovascular system function	1.75E-04	8	
**Top canonical pathways**	***P*-Value**	**Genes†**	**Ratio†**
Coagulation system	2.56E-08	(7/38)	0.184
Intrinsic prothrombin activation pathway	1.75E-04	(6/34)	0.176
Extrinsic prothrombin activation pathway	4.65E-04	(3/20)	0.15
Acute phase response signaling	5.11E-08	(15/178)	0.08

*Ingenuity® Pathway Analysis (IPA®) software was used for functional annotation and mapping of the DE genes to canonical (metabolic/regulatory) pathways, gene interaction networks, and interactive networks of transcriptional factors that regulate differential expression of target genes. The significance of representation (*P*-value) is determined by IPA based on the number of DE genes (# Genes) found in each biological category divided by the number of known genes assigned to that category by the Ingenuity® Knowledge Base [42]. The bottom panel shows the significance of the representation of DE genes in canonical pathways by IPA software. The “Genes†” and “Ratio†” columns indicate the number of observed DE genes divided by total number of genes assigned to each canonical pathway by the Ingenuity® Knowledge Base.

**Figure 2:**
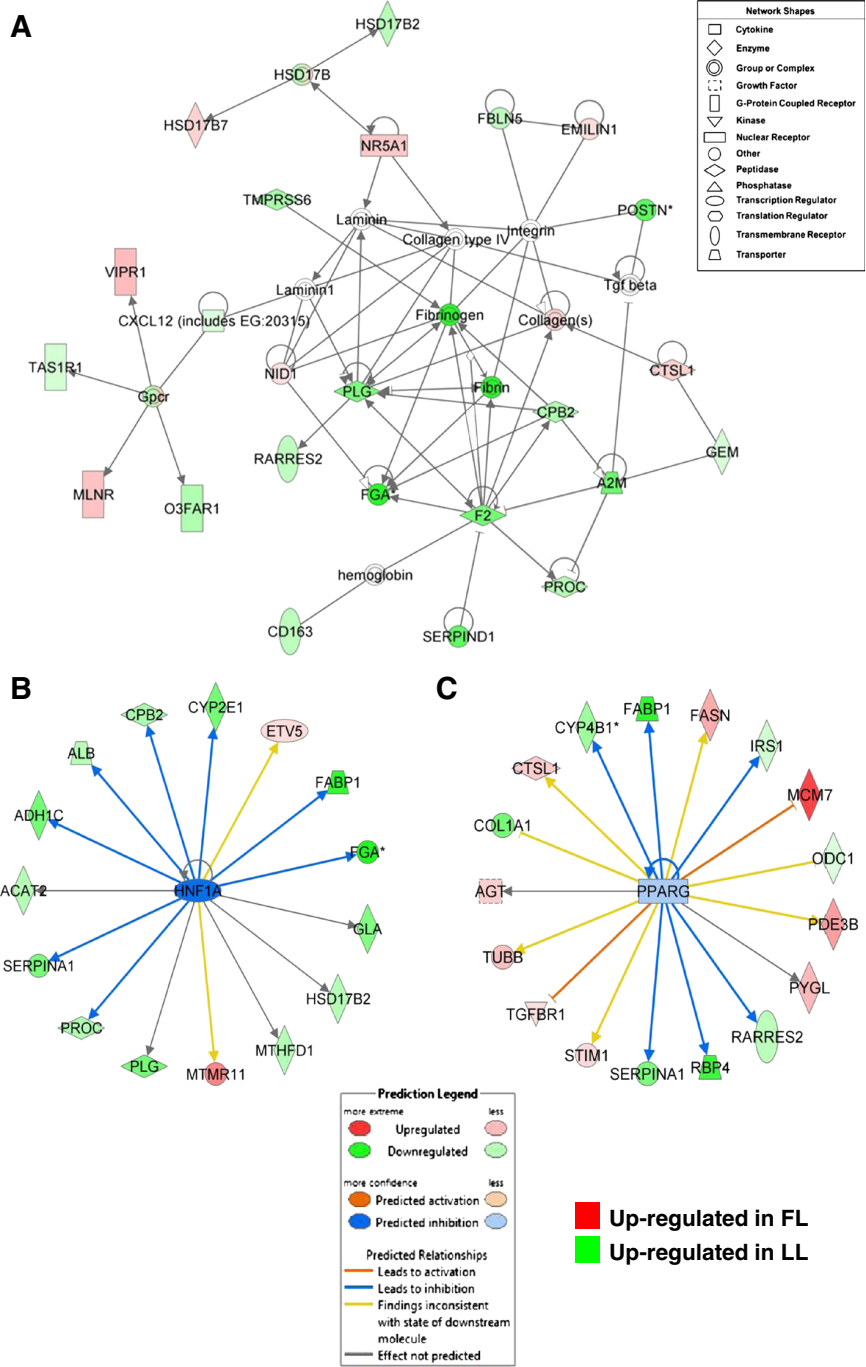
**Gene interaction network in abdominal fat of LL chickens associated with hemostasis.** Functional gene interactions networks were identified by Ingenuity Pathway Analysis (IPA**®**) software. This network shows direct gene interactions mainly in abdominal fat of LL chickens related to “Hematological System Development and Function” **(A)**. The IPA**®** Upstream Regulator Analysis identified transcription factors with direct actions on differentially expressed target genes in abdominal fat of FL and LL chickens. This analysis of upstream regulators (based on expected responses from literature and observed responses in the data set) predicts inhibition (blue color) of hepatic nuclear factor 1A (HNF1A) **(B)** and peroxisome proliferator-activated receptor gamma (PPARG) **(C)**, which would lead to inhibition (blue edges or lines) of target gene expression. Red gene symbols indicate higher expression in the FL and green gene symbols indicate higher expression in the LL.

One gene interaction network identified by IPA was heavily populated with a large number of hemostatic genes, which were up regulated in abdominal fat of the LL chickens (Figure 2). These genes are involved in coagulation [*F2*, *A2M*, carboxypeptidase B2 (*CPB2*), fibrinogen alpha (*FGA*), *PLG*, protein C (*PROC*) and serine peptidase inhibitor, clade D, member 1 (*SERPIND1*)] and inflammation [*CD163* and retinoic acid receptor responder 2 (*RARRES2*) or chemerin]. Another group of DE genes [taste receptor, type 1, member 1 (*TAS1R1*), motilin receptor (*MLNR*), vasoactive intestinal peptide receptor 1 (*VIPR1*), and omega-3 fatty acid receptor 1 (*O3FAR1*)] are G-coupled receptors linked through the chemokine ligand *CXCL12*. Three genes shown in this pathway are related to steroid metabolism [hydroxysteroid (17-β) dehydrogenase 2 (*HSD17B2*) and hydroxysteroid (17-β) dehydrogenase 7 (*HSD17B7*)] and action [nuclear receptor subfamily 5, group A, member 1 (*NR5A1*)]. The transcription factor HNF1A regulates several hemostatic genes (*FGA, PLG, PROC* and *SERPINA1*) in visceral fat of LL chickens. PPARG directly regulates three adipokines (*RARRES2, RBP4* and *SERPINA1*) expressed at higher abundance of LL and several additional genes up regulated in the FL (*AGT, CTSL1, MCM7, SIM1, TGFBR1* and *TUBB*), including three metabolic enzymes (*FASN, PDE3B* and *PYGL*).

**Figure 3:**
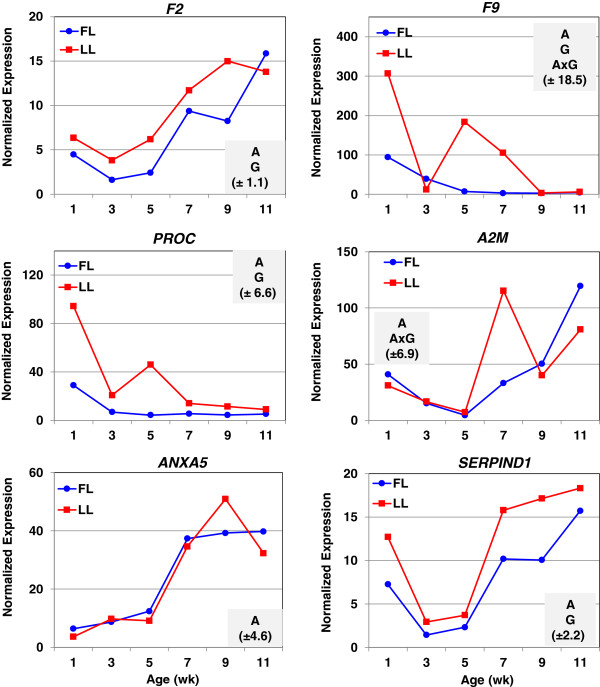
**Verification of differential expression of hemostatic genes by qRT-PCR analysis.** The abundance of six genes associated with blood coagulation was determined by quantitative reverse transcription PCR (qRT-PCR) analysis. Data points represent Least Squares Means (LSMEANS; n = 4 birds/genotype) of normalized expression values generated by the general linear models (GLM) procedure in Statistical Analysis System (SAS) software. A two-factor (genotye and age) analysis of variance (ANOVA) was used to determine significance (*P*≤0.05). The shaded box in each panel indicates significant effects of age (A), genotype (G) and/or the A x G interaction; the parenthesis shows the common standard error (SE) of LSMEANS for that gene as determined by the GLM procedure in SAS.

**Figure 4:**
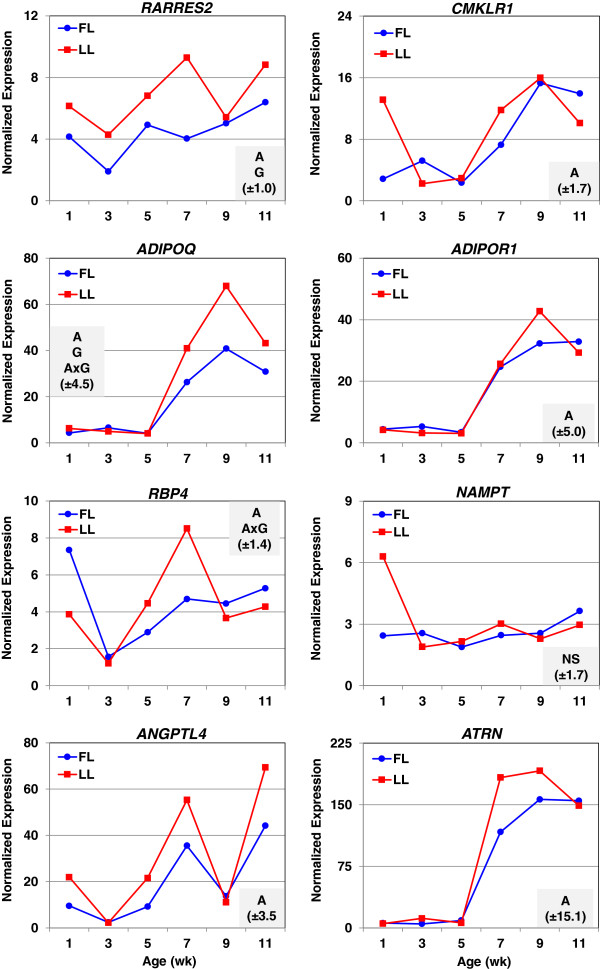
**Verification of differential expression of adipokines by qRT-PCR analysis.** The abundance of eight adipokines was determined by quantitative reverse transcription PCR (qRT-PCR) analysis. Data points represent LSMEANS (n = 4 birds/genotype) of normalized expression values. A two-factor ANOVA was used to determine significance (*P*≤0.05). The shaded box in each panel indicates significant effects of age (A), genotype (G) and/or the A × G interaction; the parenthesis shows the common standard error (SE) of LSMEANS for that gene determined by the GLM procedure in SAS.

The higher expression of select hemostatic genes found in abdominal fat of LL chickens was verified by qRT-PCR analysis (Figure 3): serine proteases [*F2*, coagulation factor IX (*F9*) and protein C (*PROC*)] and protease inhibitors [*A2M*, annexin A5 (*ANXA5*), and *SERPIND1*]. Thrombin (F2) was more abundant in abdominal fat of the LL at all ages, except 11 wk. The expression of *PROC* was 3-fold higher in the LL at 1 and 3 wk, and over 10-fold higher at 5 wk. The coagulation factor *F9* (Christmas factor) was over expressed in visceral fat of the LL by 3-fold, 24-fold, and 29-fold at 1, 5 and 7 wk, respectively. The expression patterns of two serine proteases (*F9* and *PROC*) were similar with the greatest differences at 1 and 5 wk. The qRT-PCR analysis shows similar expression patterns between some hemostatic factors and adipokines (Figure 4). For example, the expression of *ANXA5*, *F2,* adiponectin (*ADIPOQ*), adiponectin receptor 1 (*ADIPOR1*) and attractin (*ATRN*) were highest in abdominal fat of the LL at 9 wk. Similarly, expression profiles of *A2M*, retinol binding protein 4 (*RBP4*) and angiopoietin-like 4 (*ANGPTL4*) were greatest in the LL at 7 wk. The adipokine visfatin [or nicotinamide phosphoribosyltransferase (*NAMPT*)] was not differentially expressed in adipose tissue of juvenile FL and LL. Both *ADIPOQ* and *ANGPTL4* were identified in the main effect of age (A) by microarray analysis, although the log2 expression ratios were only slightly higher in the FL. The qRT-PCR analysis shows that the expression of *ADIPOQ* was higher (*P*≤0.05) in the LL between 7–11 wk of age, while the abundance of *ANGPTL4* was elevated at 1, 5, 7 and 11 wk of age, albeit only age (A) produced a significant main effect.

The top canonical pathways identified by IPA (Additional file 5) reflect the prevalence of hemostatic genes in adipose tissue of LL chickens. The IPA software provided functional assignments of DE genes to “Coagulation System” (7 genes), “Acute Phase Response Signaling” (15 genes) and “Intrinsic Prothrombin Activation” (6 genes) pathways. These adipose genes include serine proteases [*F2*, *PLG*, *PROC*, and complement factor B (*CFB*)], protease inhibitors [*A2M*, serine peptidase inhibitor clade A member 1 (*SERPINA1*), and *SERPIND1*] and transporters of retinol [retinol binding protein 4 (*RBP4*) and 7 (*RBP7*)]. The IPA functional category “Lipid Metabolism” (Additional file 6) shows high representation of numerous genes involved in “oxidation of lipid” (11/12 genes higher in LL chickens), “transport of lipid” (9/12 genes higher in LL chickens), “synthesis of lipid” (18/37 genes up regulated in FL chickens) and “metabolism of retinoid” (5/5 genes higher in LL chickens).

### Higher expression of lipogenic genes in adipose tissue of FL chickens

**Figure 5:**
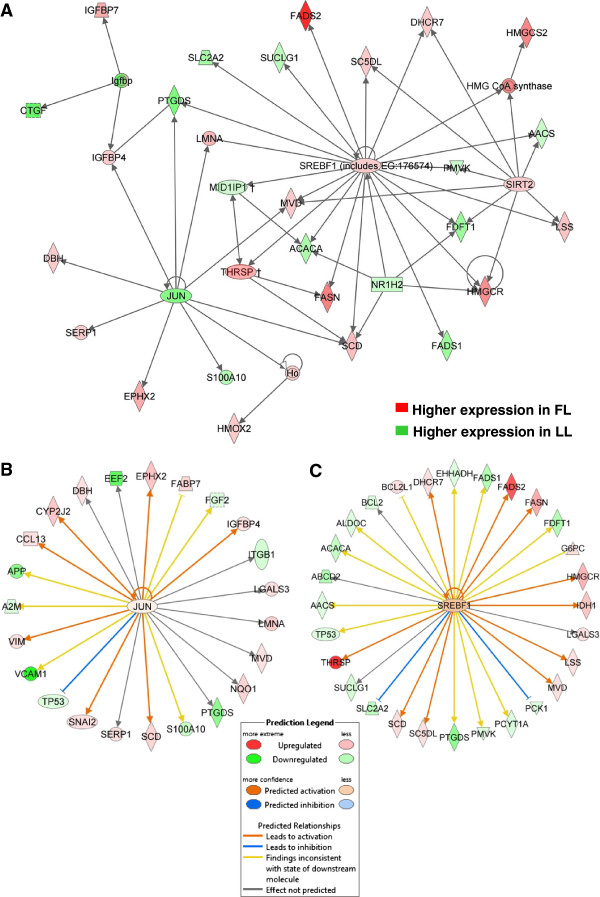
**Transcriptional regulation of gene interaction network in abdominal fat of FL and LL chickens controlling lipogenesis.** Functional gene interactions and up-stream regulators were identified by IPA (gene symbols and color schemes as described in Figure 2). Direct interactions (solid lines) were found among transcription regulators [*JUN, SREBF1*, *SIRT2, MID1IP1, NR1H2* (*LXRB*) and *THRSP*] and lipogenic genes **(A)**. ✝THRSP and its paralog MID1IP1 (or THRSP-like, *THRSPL*) were not con sidered as transcription regulators by IPA software. *THRSPA* was added to this network based on microarray and qRT-PCR analysis (Additional files 3 and 7) and known involvement of THRSP in regulating expression of lipogenic enzymes across multiple species of birds and mammals. This analysis of upstream regulators predicts activation of JUN **(B)** and sterol response element binding factor 1 (SREBF1) **(C)**, which would lead to inhibition or activation [orange edges (lines)] expression of DE target genes. Gene symbol color indicates higher expression in the FL (red) or higher expression (green) in the LL.

The abdominal fat of FL chickens exhibits higher expression of lipogenic transcription factors [sterol regulatory element binding transcription factor 1 (*SREBF1*), thyroid hormone responsive Spot 14 protein (*THRSP*) and sirtuin 2 (*SIRT2*)] (Figure 5A). In contrast, additional regulators of transcription [THRSP-like (THRSPL) or MID1 interacting protein 1 (*MID1IP1*); the nuclear liver X receptor-β (*LXR*β or *NR1H2*); and the proto-oncogene *jun* (*JUN*)] were more abundant in abdominal fat of the LL. As shown in this IPA gene interaction network, *SREBF1* directly up regulates several genes in the FL that are involved in lipid biosynthesis [*FASN*, stearoyl-CoA desaturase (*SCD*), fatty acid desaturase 2 (*FADS2*), sterol-C5-desaturase (*SC5DL*), mevalonate decarboxylase (*MVD*), 7-dehydrocholesterol reductase (*DHCR7*), 3-hydroxy-3-methylglutaryl-CoA reductase (*HMGCR*) and lanosterol synthase (*LSS*)] and ketogenesis [3-hydroxy-3-methylglutaryl-CoA synthase 2 (*HMGCS2*)]. Some of these genes are also targets of *SIRT2* and *THRSPA* and differentially expressed in adipose tissue of the FL. In addition, *SREBF1* directly affects numerous genes that are expressed higher in the LL [fatty acid desaturase 1 (*FADS1*), acetyl-CoA carboxylase alpha (*ACACA*), acetoacetyl-CoA synthetase (*AACS*), farnesyl-diphosphate farnesyltransferase 1 (*FDFT1*), solute carrier family 2 (*SLC2A2*; or facilitated glucose transporter 2, *GLUT2*), succinate-CoA ligase, alpha subunit (*SUCLG1*), and phosphomevalonate kinase (*PMVK*)]. Two *JUN* targets, prostaglandin D2 synthase (*PTGDS*) and *MID1IP1* (which regulates transcription of *ACACA*), were over-expressed in adipose tissue of the LL. Insulin-like growth factor binding protein 4 (*IGFBP4*) is another target of *JUN* that was expressed at higher levels in FL adipose tissue. The IPA Upstream Regulator Analysis predicts that JUN and SREBF1 lead to activation (indicated by orange arrows) of numerous up-regulated target genes (red symbols) in abdominal fat of the FL chickens (Figure 5B and 5C). In contrast, the blue blunted lines show predicted inhibition and down-regulation of DE genes by JUN (*TP53*) and SREBF1 (*PCK1* and *SLC2A2*) in the LL (green symbols). The yellow arrows indicate that the IPA analysis would expect these targets to be activated by JUN and SREBF1, rather than down regulated as shown by these green gene symbols. The majority of the up-regulated genes found in abdominal fat of the FL (red symbols) are enzymes involved in lipid metabolism.

**Figure 6:**
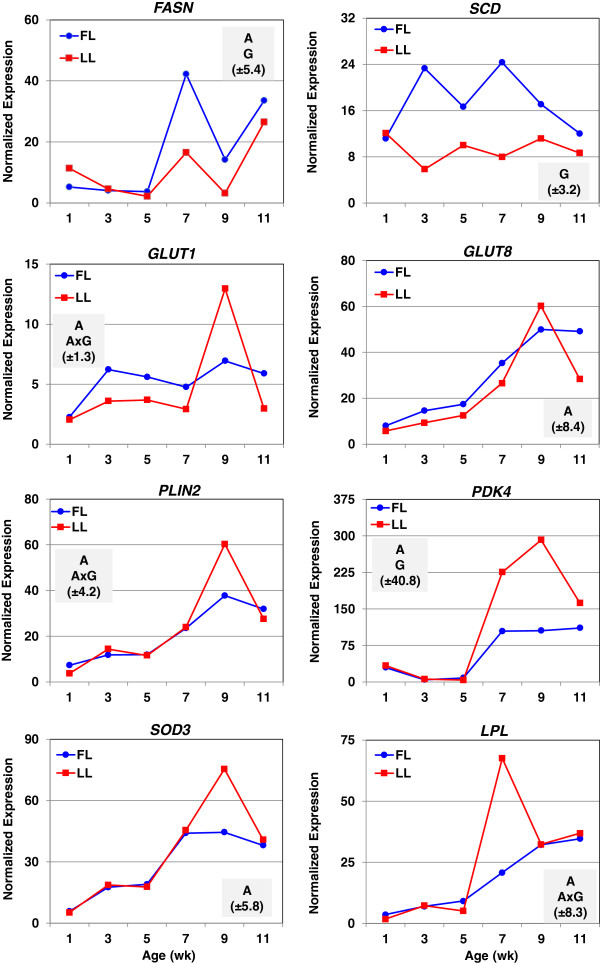
**Expression of genes associated with lipid metabolism by qRT-PCR analysis.** mRNA expressions of eight genes involved in lipid metabolism were determined by quantitative reverse transcription PCR (qRT-PCR). Each data point represents LSMEANS (n = 4 birds/genotype) of normalized expression values. A two-factor ANOVA was used to determine significance (*P*≤0.05). The shaded box in each panel indicates significant effects of age (A), genotype (G) and/or the A × G interaction; the parenthesis shows the common standard error (SE) of LSMEANS for that gene determined by the GLM procedure in SAS.

The expression profiles of eight genes mainly associated with lipid metabolism were examined by qRT-PCR analysis (Figure 6). A main effect (*P*≤0.05) of genotype (G) was observed for *FASN* (4-fold increase in FL at wk 7), *SCD* (4-fold and 3-fold increase in FL at wk 3 and 7, respectively), and pyruvate dehydrogenase kinase, isozyme 4 (*PDK4*, over expressed in LL chickens from 7 to 11 wk). A significant age by genotype (A x G) interaction (*P*≤0.05) was observed for facilitated glucose transporter, member 1 (*GLUT1*), perilipin 2 (*PLIN2*) and lipoprotein lipase (*LPL*). A main effect of age (A; *P*≤0.05) was also observed for *FASN*, *GLUT1, PLIN2, PDK4, LPL*, facilitated glucose transporter, member 8 (*GLUT8*) and superoxide dismutase 3 (*SOD3*).

**Figure 7:**
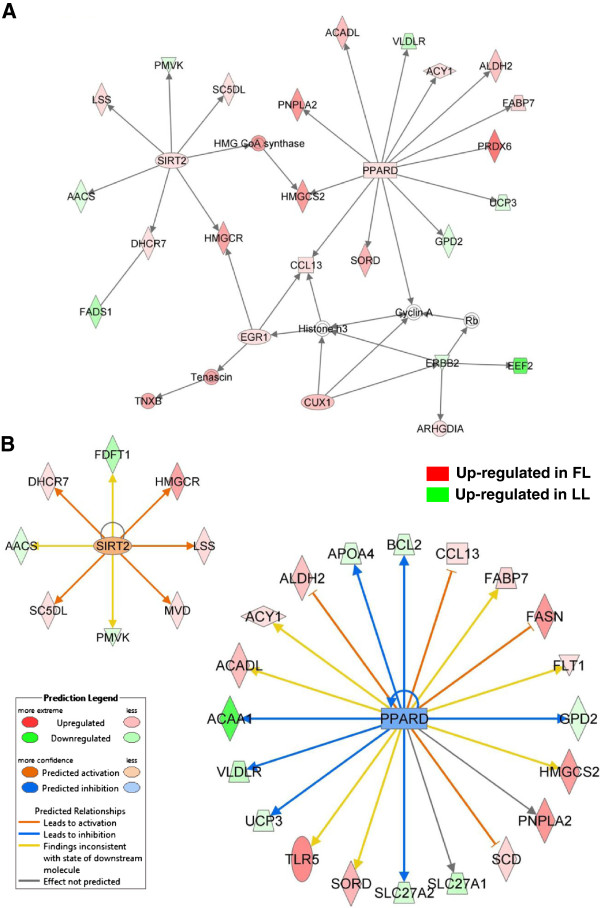
**Transcriptional regulators of DE genes controlling lipogenesis in abdominal fat of FL and LL chickens.** A large number of DE lipogenic genes interact with two transcriptional regulators, *SIRT2* and *PPARD***(A)**. The IPA Upstream Regulator Analysis **(B)** predicts that the up-regulation of *SIRT2* leads to activation of five lipogeneic genes (orange-edged arrows), whereas, the predicted inhibition of PPARD would lead to down regulation (blue-edged arrows) of seven DE target genes in the FL [or up-regulation (green gene symbols) in the LL]. The predicted activation of major lipogenic genes (*ALDH2, CCL13, FASN* and *SCD*) would be blocked (blunt orange arrow) by PPARD.

Another network populated by numerous DE genes (up regulated in the FL) that control lipid metabolism shows the interaction of four transcription regulators (*SIRT2, PPARD, EGR1* and *CUX1*), also up regulated in visceral fat of the FL (Figure 7A). Peroxisome proliferator-activated receptor delta (*PPARD*) interacts directly with patatin-like phospholipase domain containing 2 (*PNPLA2*), long chain acyl-CoA dehydrogenase (*ACADL*), aminoacylase 1 (*ACY1*), aldehyde dehydrogenase 2 (*ALDH2*), peroxiredoxin 6 (*PRDX6*), fatty acid binding protein 7 (*FABP7*), sorbitol dehydrogenase (*SORD*) and chemokine (C-C motif) ligand 13 (*CCL13*). Early growth response 1 (*EGR1*) interacts with *CCL13* and 3-hydroxy-3-methylglutaryl CoA reductase (*HMGCR*), the rate-limiting enzyme in biosynthesis of cholesterol, which is a target of the histone deacetylase sirtuin 2 (SIRT2). The ketogenic enzyme 3-hydroxy-3-methylglutary-Coenzyme A synthase 2 (*HMGCS2*) is a downstream target of both SIRT2 and PPARD. Three additional metabolic enzymes [lanosterol synthase (LSS) and 7-dehydrocholesterol reductase (DHCR7)] were also expressed at higher levels in the FL, while acetoacetyl-CoA synthetase (*AACS*), fatty acid desaturase 1 (*FADS1*), and phosphomevalonate kinase (*PMVK*) were more abundant in abdominal fat of the LL. The Ingenuity Upstream Regulator Analysis identified several additional targets of PPARD (Figure 7B), which were expressed at higher levels in either the FL (*FASN*, *FLT1, SCD* and *TLR5*) or the LL (*ACAA, APOA4, BCL2, GPD2, SLC27A1, SLC27A2, UCP3* and *VLDLR*) chickens. The IPA software predicts that PPARD is inhibited (blue color) based on prior knowledge of PPARD action in mammals and the observed higher expression of PPARD-activated targets in the LL (green symbols). IPA predicts that SIRT2 should be activated (orange color) and has a direct positive action on five target genes (*DHCR7, HMGCR, LSS, MVD* and *SC5DL*), which were up regulated in adipose tissue of the FL chickens. However, the yellow-colored arrows indicate that IPA expected three target genes (*AACS, FDFT1* and *PMVK*) to be up regulated in the FL, rather than the LL as we observed.

### Ligand activated nuclear receptors and other transcription factors

Of special interest are genes involved in ligand-activated gene transcription (e.g., retinol and thyroid hormone signaling) which regulate lipid metabolism (Table 3). Functional annotation of DE genes by IPA analysis identified five genes [alcohol dehydrogenase 1C (*ADH1C*)*,* alcohol dehydrogenase 5 (*ADH5*)*,* cytochrome P450, family 2, subfamily E, polypeptide 1 (*CYP2E1*)*, RARRES2* and *RBP4*] related to “metabolism of retinoid” (Additional file 6). An additional four retinol-related genes [*RBP7,* nucleolar protein 7 (*NOL7*)*,* transthyretin (*TTR*) and retinol dehydrogenase 1 (*RDH1*)] were found by microarray analysis (Additional file 3).

**Table 3:** Functional categories of DE and prior candidate genes expressed in abdominal fat and the average fold change (FL/LL) as determined by microarray and/or qRT-PCR analyses

**Functional category**	**Symbol**	**Gene name**	Microarray (FC)*	qRT-PCR (FC)*
***Hemostasis***	*A2M*	Alpha-2-macroglobulin	−1.89	−1.10
	*AGT*	Angiotensinogen	1.20	-
	*ANG*	Angiogenin	−2.51	-
	*CFB*	Complement factor B	−1.49	-
	*CPB2*	Carboxypeptidase B2	−1.43	-
	*CPM*	Carboxypeptidase M	−1.32	-
	*F2*	Thrombin	−1.85	−1.35
	*F9*	Christmas factor	−1.51	−4.04
	*FGA*	Fibrinogen alpha	−2.61	-
	*PLG*	Plasminogen	−1.79	-
	*PROC*	Protein C	−1.39	−3.54
	*SERPINA1*	Antitrypsin	−1.75	-
	*SERPIND1*	Heparin cofactor	−2.00	−1.50
	*THBS2*	Thrombospondin 2	−1.17	-
***Adipokines***	*ADIPOQ*	Adiponectin	1.03	−1.48
	*ANGPTL4*	Angiopoietin-like 4	1.01	−1.58
	*ATRN*	Attractin	−1.12	−1.22
	*CFD*	Adipsin	1.24	-
	*LPL*	Lipoprotein lipase	-	−1.41
	*NAMPT*	Visfatin	-	−1.20
	*RARRES2*	Chemerin	−1.32	−1.54
	*RBP4*	Retinol binding protein 4	−2.33	−1.11
***Lipogenesis***	*DHCR7*	7-Dehydrocholesterol reductase	1.11	-
	*FADS2*	Fatty acid desaturase 2	1.21	-
	*FASN*	Fatty acid synthase	1.36	1.60
	*G6PC*	Glucose-6-phosphatase, catalytic subunit	1.46	-
	*scGH*	Growth hormone, chicken, short form	1.15	-
	*HMGCR*	3-Hydroxy-3-methylglutaryl-CoA reductase	1.09	-
	*INSIG2*	Insulin induced gene 2	1.74	-
	*LCAT*	Lecithin-cholesterol acyltransferase	1.32	-
	*MVD*	Mevalonate (diphospho) decarboxylase	1.20	-
	*SCD*	Stearoyl-CoA desaturase	1.48	1.88
	*SREBF1*	Sterol regulatory element binding transcription factor 1	1.12	1.32
	*THRSPA*	Thyroid hormone responsive spot 14 A	-	1.64
	*TXNIP*	Thioredoxin interacting protein	-	1.80
***Lipolysis***	*ACAT1*	Acetyl-CoA acetyltransferase 1	−3.18	-
	*ADH1C*	Alcohol dehydrogenase 1C (class I), gamma polypeptide	−1.81	-
	*APOA1*	Apolipoprotein A-I	−1.16	-
	*APP*	Amyloid beta (A4) precursor protein	−1.15	-
	*BCMO1*	beta-carotene 15,15′-monooxygenase	-	−1.13
	*BCO2*	beta-carotene oxygenase 2	−1.15	−1.48
	*CYP27A1*	Cytochrome P450, family 27, subfamily A, polypeptide 1	−1.14	-
	*CYP2E1*	Cytochrome P450, family 2, subfamily E, polypeptide 1	−1.80	-
	*EHHADH*	Enoyl-CoA, hydratase/3-hydroxyacyl CoA dehydrogenase	−1.09	-
	*GAMT*	Guanidinoacetate N-methyltransferase	−1.21	-
	*HADHB*	Hydroxyacyl-CoA dehydrogenase (trifunctional protein)	−1.10	-
	*HSD17B4*	Hydroxysteroid (17-beta) dehydrogenase 4	−1.92	-
	*HSD17B6*	Hydroxysteroid (17-beta) dehydrogenase 6	−1.19	-
	*IRS1*	Insulin receptor substrate 1	−1.59	-
	*PDK4*	pyruvate dehydrogenase kinase, isozyme 4	-	−1.99
	*PHYH*	Phytanoyl-CoA 2-hydroxylase	−1.56	-
	*SLC2A2*	Facilitated glucose transporter 2 (*GLUT2*)	−2.23	-
	*SOD3*	superoxide dismutase 3, extracellular	−1.10	−1.20
	*TP53*	Tumor protein p53	−1.29	-
	*UCP3*	Uncoupling protein 3 (mitochondrial, proton carrier)	−1.21	-

*Fold-change (FC) represents the ratio of FL/LL transcript abundance averaged across six juvenile ages (1–11 wk). Prior candidate genes were identified as differentially expressed (DE) genes by previous microarray or qRT-PCR analysis of previous genetic, nutritional or hormonal perturbation studies. Pearson’s correlation coefficient (r) of expression ratios (FL/LL) of 15 select genes subjected to both microarray and qRT-PCR analyses indicates a significant (*P*≤0.01) correlation between the two methods (r = 0.64). The exclusion of two genes with the lowest microarray FC estimate (*ANGPTL4* and *ADIPOQ*) greatly increases the Pearson correlation coefficient (r = 0.79) and the significance level (*P*≤0.001).

The qRT-PCR analysis of six candidate transcription factors is presented in Additional file 7. Four genes [peroxisome proliferator-activated receptor alpha (*PPARA*), peroxisome proliferator-activated receptor gamma (*PPARG*), *PPARD* and sterol regulatory element binding transcription factor 2 (*SREBF2*) showed only a main effect of age (A). A main effect of genotype (G) was observed for *SREBF1* due to higher expression in the FL at 1 and 5 wk. Similarly, the abundance of retinoid X receptor, gamma (*RXRG*) was higher in adipose tissue of the FL at 1, 5 and 11 wk, which produced a main effect of genotype (G).

The abundance of eight additional genes controlling metabolism and signaling of thyroid hormone and retinol was examined by qRT-PCR analysis (Additional file 8). Type I iodothyronine deiodinase (*DIO1*), which converts the prohormone T_4_ to metabolically active T_3_, showed only a main effect of age, whereas type III iodothyronine deiodinase (*DIO3*) presented main effects of age and genotype due to a consistently higher abundance in abdominal fat of LL chickens (Additional file 8-A). In contrast, the transcriptional regulator *THRSPA* and thioredoxin interacting protein (*TXNIP*) showed a main effect of age (A) and genotype (G) with higher expression in visceral fat of FL chickens at five of the six ages examined. Four genes involved in retinol metabolism [beta-carotene 15, 15′-monooxygenase (*BCMO1*), beta-carotene oxygenase 2 (*BCO2*), retinol saturase (*RETSAT*)] and the retinoic acid-induced gene 3 (*RAIG3*) [or G protein-coupled receptor, family C, group 5, member C (*GPRC5C*)] were also examined by qRT-PCR analysis (Additional file 8-B). Although higher in the LL between 7 and 11 wk of age, *BCMO1* produced only a main effect of age (A). The expression of *BCO2* sharply increased with age (main effect) and was consistently higher in abdominal fat of the LL birds (main effect of genotype). Similarly, *RAIG3* showed main effects of age and genotype, with higher expression in the LL at 7 wk of age. The abundance of *RETSAT* was higher in visceral fat of the FL at 3 and 9 wk. Furthermore, the retinoid ligand-activated transcription factor *RXRG* was up-regulated in the FL, especially at 11 wk of age (Additional file 7).

An array of DE and prior candidate genes was selected for verification of gene expression using qRT-PCR analysis (Table 3; Figures 3, 4 and 6; Additional files 7 and 8). Pearson’s correlation coefficient (r) of expression ratios (FL/LL) of 15 select genes subjected to both microarray and qRT-PCR analyses indicates a significant (*P*≤0.01) correlation between the two methods (r = 0.64). The exclusion of two genes with the lowest microarray FC estimate (*ANGPTL4* and *ADIPOQ*) greatly increased the Pearson correlation coefficient (r = 0.79) and the significance level (*P*≤0.01).

**Figure 8:**
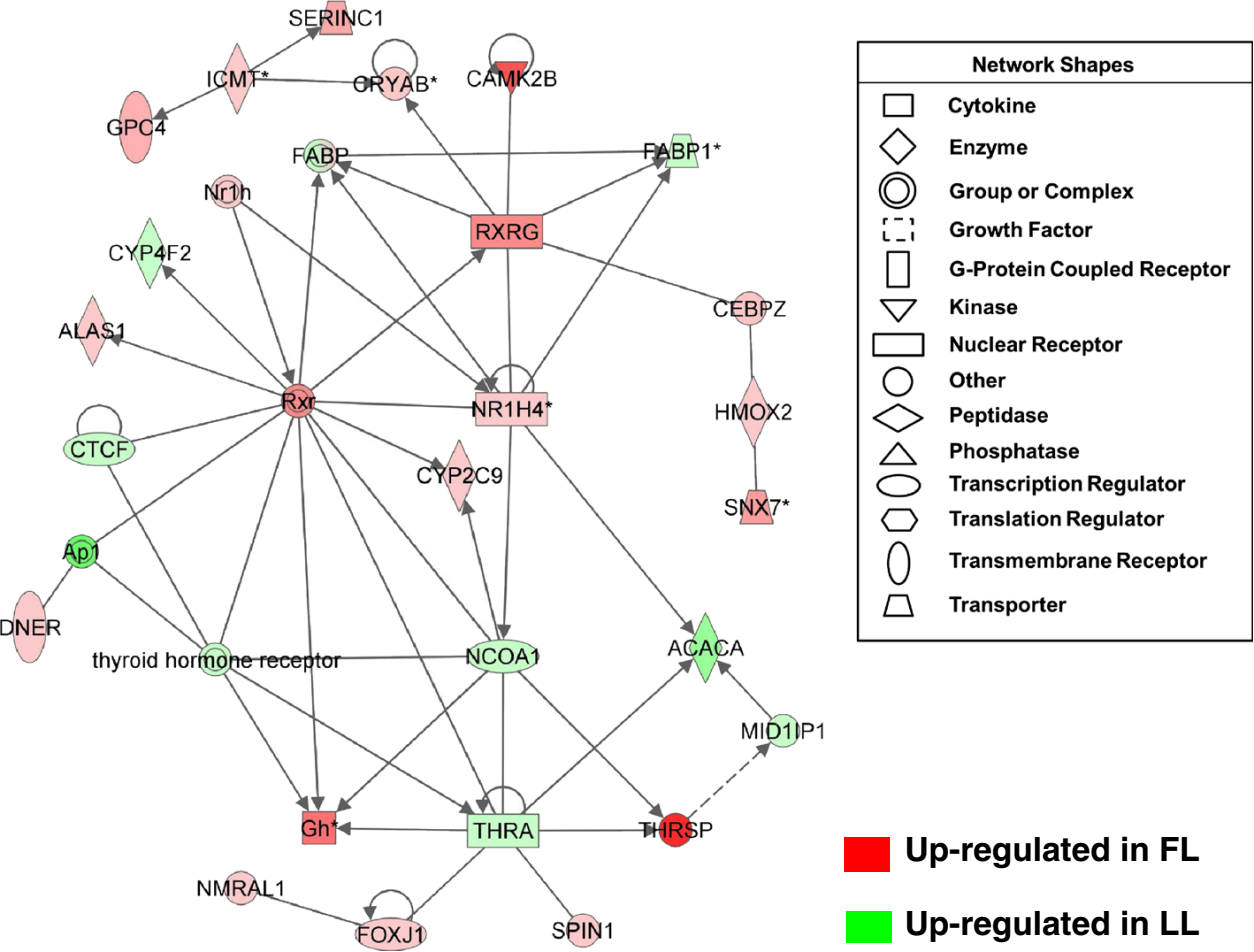
**Gene interaction network of nuclear receptors, co-activators and regulators of gene transcription in abdominal fat of juvenile FL and LL chickens.** This gene network shows direct interactions of seven transcriptional regulators [*CEBPZ, RXRG, NR1H4* or farnesoid X receptor (*FXR*), *NCOA1* or steroid receptor coactivator 1 (*SRC-1*), *THRA, THRSP* and *MID1IP1* (or THRSP-like, *THRSPL*)] and their target genes. Gene symbol color indicates higher expression in the FL (red) or higher expression (green) in the LL.

Another gene interaction network (Figure 8) identified by IPA shows interactions of several ligand-activated nuclear receptors and transcription regulators [*RXRG, CEPBZ, NR1H4* (farnesoid X receptor, FXR), *THRA, THRSP, MID1IP1*, nuclear receptor coactivator 1 (*NCOA1*), forkhead box J1 (*FOXJ1*), and CCCTC-binding factor (*CTCF*)]. The target genes of these upstream regulators were up regulated in abdominal fat of the FL [*GH, DNER, CYP2C9, ALAS1, CRYAB, ICMT, GPC4, SERINC1, CAMK2B, HMOX2* and *SNX7*] or LL chickens (*CYP4F2, FABP1* and *ACACA*).

**Figure 9:**
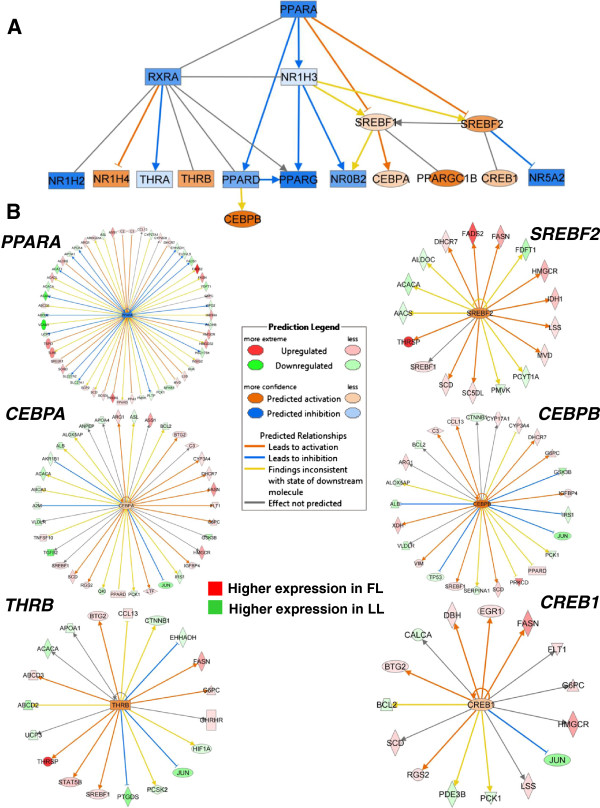
**Upstream regulators of gene transcription in abdominal fat of juvenile FL and LL chickens.** Ingenuity® Upstream Regulator Analysis revealed a large number of transcriptional regulators (see Table 4) controlling lipid metabolism genes in abdominal fat **(A)**. This IPA analysis shows “up-stream regulators” and their downstream targets found among DE fatty acid metabolism genes identified in abdominal fat of the FL and LL chickens. Differentially expressed gene targets regulated by six additional transcription factors are shown **(B)**. The IPA prediction of activation (orange lines and symbols) or (blue lines and symbols) inhibition states is based on prior knowledge accrued by Ingenuity® Knowledge Base and expression values of differentially expressed genes identified by microarray analysis of abdominal fat in juvenile FL and LL chickens. Gene symbol color indicates higher expression in the FL (red) or higher expression (green) in the LL.

A final non-redundant set of genes involved in lipid metabolism was identified by IPA from the G, A and A × G DE gene lists and then was used for Ingenuity® Upstream Regulator Analysis. This analysis illustrates the interaction of numerous ligand-activated nuclear receptors and other transcription factors (TF), providing predictions of either an activated (orange color) or inhibited (blue color) state (Figure 9). These predictions are based on prior knowledge of transcriptional responses (from human and murine studies in the literature) and the responses of downstream targets found in the DE gene data set. For example, this mechanistic network of transcription regulators indicates whether the TF (orange color) and target gene (red gene symbol) are both activated or if the activity of the TF is inhibited (blue color), which would be associated with increased expression in the LL (green gene symbol). This mechanistic network predicts inhibition (blue lines and symbols) of eight transcription factors (PPARA, RXRA, NR1H2, NR1H3, PPARD, PPARG, NROB2 and NR5A2) and the activation (orange lines and symbols) of an additional eight transcription factors (NR1H4, THRB, CEBPA, CEBPB, CREB1, PPARGC1B, SREBF1 and SREBF2) (Table 4). The gene targets are presented for two transcription factors (PPARA and CEBPA) predicted to be inhibited and four transcription factors (THRB, SREBF2, CEBPB and CREB1) that were predicted to be activated by the IPA Upstream Regulator Analysis. This mechanistic analysis shows that three transcription factors (PPARA, CEBPA and CEBPB) exert direct actions on target genes up-regulated in the LL, while three other transcription factors (THRB, SREBF2 and CREB1) mainly target up-regulated genes in the FL, which are involved in the synthesis, transport or metabolism of lipids.

**Table 4:** Transcriptional regulators of genes that control the divergence of abdominal fatness in FL and LL chickens

**Symbol**	**NCBI Entrez gene name**	**Activation z-score**	**P-value of overlap**	**# Target molecules**
CEBPA	CCAAT/enhancer binding protein (C/EBP), alpha	0.379	1.61E-15	34
CEBPB	CCAAT/enhancer binding protein (C/EBP), beta	1.935	1.75E-10	25
CREB1	cAMP responsive element binding protein 1	0.527	1.64E-05	15
NR0B2	nuclear receptor subfamily 0, group B, member 2 (SHP)	−0.84	3.74E-08	10
NR1H2	nuclear receptor subfamily 1, group H, member 2 (LXRB)	−1.512	2.64E-10	12
NR1H3	nuclear receptor subfamily 1, group H, member 3 (LXRA)	−0.2	4.69E-11	14
NR1H4	nuclear receptor subfamily 1, group H, member 4 (FXR)	1.076	1.40E-06	11
NR5A2	nuclear receptor subfamily 5, group A, member 2 (LRH1)	−1.412	3.57E-04	7
PPARA	peroxisome proliferator-activated receptor alpha	−1.339	1.78E-32	54
PPARD	peroxisome proliferator-activated receptor delta	−0.767	3.13E-13	21
PPARG	peroxisome proliferator-activated receptor gamma	−1.629	1.38E-27	48
PPARGC1B	peroxisome proliferator-activated receptor gamma, coactivator 1 beta	1.488	1.37E-12	12
RXRA	retinoid X receptor, alpha	−0.932	3.32E-18	31
SREBF1	sterol regulatory element binding transcription factor 1	0.511	2.91E-20	29
SREBF2	sterol regulatory element binding transcription factor 2	1.171	2.78E-15	17
THRA	thyroid hormone receptor, alpha	−0.246	2.08E-08	12
THRB	thyroid hormone receptor, beta	1.135	7.18E-11	19

Ingenuity Upstream Regulator Analysis® identified multiple transcription factors (mainly ligand-activated nuclear factors) controlling a greatly amplified number of lipid metabolism genes in abdominal fat during juvenile development (1–11 wk) of FL and LL chickens (see Figure 9). The activation Z-score indicates whether the observed gene responses to upstream regulators agrees with expected changes derived from the literature and accrued in the Ingenuity® Knowledge Base [42]. A Fisher’s Exact Test was used to determine the significance for enrichment of our target DE genes controlled by numerous upstream regulators and annotated in the Ingenuity® Knowledge Base.

*Abbreviations*: small heterodimer partner (SHP), liver X receptor alpha (LXRA), liver X receptor beta (LXRB), farnesoid X nuclear receptor (FXR), and liver receptor homolog 1 (LRH1).

## Discussion

The divergent FL and LL chickens were originally developed as experimental models to study genetic and endocrine mechanisms controlling excessive abdominal fatness [43]. Indeed, juvenile FL and LL chickens exhibit a 2.5-fold difference in abdominal fatness between 3 and 11 wk of age while maintaining similar rates of growth (Table 1), feed intake, and energy metabolism [44]. The divergence of adiposity between the FL and LL chickens occurs at 3 wk of age [27]; hyperplasia of adipocytes was found as early as 2 wk of age in the FL [28], followed by marked hypertrophy of adipocytes by 9 wk of age [27]. The FL chickens appear to favor partitioning of energy and nutrients into abdominal fat, whereas the LL deposit more protein into skeletal muscle, especially breast muscle [44]. A consistent feature of metabolism in the FL chickens is a glucose-insulin imbalance, where plasma glucose levels are lower and insulin levels are slightly elevated [11,27]. The higher rate of lipogenesis observed in liver of FL chickens contributes to a greater accumulation of abdominal fat in this genotype [45,46]. The search for major genes contributing to the divergence in adiposity between the FL and LL has primarily focused on the liver [3,19,29-32]. In the present study, the Del-Mar 14K Integrated Systems microarray was used to examine gene expression profiles of abdominal fat in juvenile FL and LL cockerels across six ages (1–11 wk). This analysis of time-course transcriptional profiles has provided the first panoramic view of the abdominal fat transcriptome in the FL and LL chickens and given functional insight into the 2.5-fold divergence of adiposity. In particular, we have discovered numerous DE genes that are involved in hemostasis (blood coagulation), adipokine signaling, thyroid hormone and retinol action, and lipogenesis in abdominal fat of FL and LL chickens during juvenile development. These findings are unlike previous microarray studies of adipose tissue in meat-type chickens.

### Higher expression of blood coagulation factors in adipose tissue of LL chickens

A large number of genes involved in hemostasis were differentially expressed in adipose tissue of LL chickens (Table 3). Several coagulation factors identified in our transcriptional analysis of adipose tissue are either proteases (*i.e*., *F2*, *F9, PLG*, *PROC*, and *CFB*) or protease inhibitors (*i.e*., *A2M*, *ANXA5, SERPINA1*, and *SERPIND1*). We also found higher expression of carboxypeptidases [carboxypeptidase B2 (*CPB2* or thrombin-activatable fibrinolysis inhibitor) and carboxypeptidase M (*CPM*)] in abdominal fat of LL chickens. Our discovery of a higher abundance of several genes encoding blood clotting factors in LL chickens is quite peculiar given that fattening, rather than leanness, in mammals is usually associated with the prothrombotic state [47-50]. In fact, obesity in humans is described as chronic low-grade inflammation where expression of hemostatic genes [e.g., serine peptidase inhibitor, clade E (nexin, plasminogen activator inhibitor type 1), member 1 (*PAI-1*), thrombin, fibrinogen and von Willebrand factor (VWF)] are positively associated with greater deposition of adipose tissue [47,51]. The adipokine *PAI-1* (*SERPINE1*) encodes a secreted regulator of fibrinolysis, which serves as a biomarker for metabolic syndrome in humans [51]. Although PAI-1 has not been mapped to the chicken genome, we did find higher expression of the plasminogen activator inhibitor 1 RNA-binding protein (*SERBP1*) in abdominal fat of the FL chickens. The SERBP1 protein greatly increases the degradation of *PAI-1* mRNA in rat hepatoma cells [52]. In addition, SERBP1 functions as a partner with the progesterone receptor membrane component 1 (PGRMC1) in mediating the anti-apoptotic action of progesterone on the female reproductive tract of humans [53]. Our identification of *SERBP1* and its up-regulation in adipose tissue of the FL suggest that a functional homolog of *PAI-1* does exist in the chicken. Another related member of the same clade as *PAI-I*, *SERPINE2* was not differentially expressed in abdominal fat of FL and LL chickens according to microarray analysis. Since *SERPINE2* was one of the most stably expressed genes in our qRT-PCR analysis, it was used as a housekeeping gene to normalize gene expression. Another hemostatic gene up regulated in abdominal fat of LL chickens was thrombospondin 2 (*THBS2*), which inhibits adipogenesis in mammals [54].

Hemostatic proteins have several functions, some of which include removal of signal peptides, activation of zymogens, transport of enzymes, or degradation of active enzymes. Given that many adipokines have a high functional and structural similarity to the classic coagulation factors and other hemostatic factors (e.g., *ANGPTL4* contains a fibrinogen-like domain), it is reasonable to assume that these proteases act on pre-pro-adipokines or other secreted proteins expressed in adipose tissue. Little is known about the expression of blood coagulation genes in visceral fat or their role in the development of adiposity in chickens. Using K-means clustering (data not shown), we found that the expression profiles for most genes involved in coagulation were clustered with those of adipokines; this general trend was verified by qRT-PCR analysis (Figures 3 and 4). For example, secreted proteins *ADIPOQ* and *ATRN* have expression patterns that are similar to those of serine proteases (e.g., *F2*) and protease inhibitors (e.g., *ANXA5*). Further, the developmental profiles of *RBP4* and *ANGPTL4* were similar to that of *A2M,* a protease inhibitor and transporter of cytokines. The positive correlation of expression patterns between coagulation factors and adipokines is not surprising given that many adipokines are associated with hemostatic or inflammatory processes (e.g. *RARRES2*); and conversely, several genes involved in coagulation are considered as adipokines (e.g. *PAI-1, A2M, F2* and *FGA*). Furthermore, a similar transcriptional analysis of liver from the same individual FL and LL birds failed to reveal differential expression of these blood coagulation factors [3]. The lack of a parallel effect of genotype on hepatic expression of coagulation factors in the FL and LL chickens suggests that their ectopic expression in abdominal fat is specific and without consequence to systemic hemostasis.

### Adipokines identified in abdominal fat of FL and LL chickens

A prime example of proteolytic processing of adipokines comes from our discovery of chemerin [or *RARRES2* (retinoic acid receptor responder, tazarotene induced, 2)], which is expressed at higher levels in abdominal fat of LL chickens. Chemerin is a recently discovered adipokine that regulates adipogenesis; and chemerin can be transformed into a pro-inflammatory protein, a cell adhesion factor or an anti-inflammatory peptide, depending upon cleavage by specific proteases [55-57]. After removal of the N-terminal signal peptide, pro-chemerin is processed at the C-terminal end by serine proteases to generate an active pro-inflammatory adipokine, which can be cleaved further at its C-terminal end by cysteine proteases to generate an anti-inflammatory peptide [58]. Active chemerin appears to exert its action by binding its extracellular receptor *CMKLR1* on adipocytes and/or *CCRL2* on activated macrophages, which then forms an adhesive bridge between these two resident cells in adipose tissue during the inflammatory response [58]. Adipocyte-derived chemerin causes insulin resistance in skeletal muscle cells [59]; and as a secreted adipokine, chemerin regulates myogenesis by providing negative cross-talk between adipose tissue and skeletal muscle [60]. Consequently, chemerin functions as a chemokine for leukocytes, an adipokine that regulates angiogenesis, and a biomarker of metabolic syndrome and obesity in humans [61-63]. However, chemerin appears to be associated with leanness in the chicken.

Retinol binding protein 4 (*RBP4*), the main transporter of retinol in blood, is another adipokine that was expressed higher in abdominal fat of LL chickens at 5 and 7 wk of age. Like chemerin, RBP4 serves as a biomarker of obesity-related diseases including insulin resistance, dyslipidemia, hypertension, and visceral obesity in both adult and adolescent humans [64]. Similar to other genes involved in coagulation, chemerin and *RBP4* are expressed higher in LL, rather than FL chickens. In contrast, several adipokines (*ADIPOQ*, *ADIPOR1* and *ATRN*) found in abdominal fat of FL and LL chickens are regulated similar to mammals. For example, *ADIPOQ* is inversely related to fatness and it is associated with increased insulin sensitivity in mammals [65]. From the qRT-PCR analysis, we observed late up-regulation of *ADIPOQ* (wk 7–11) and its receptor *ADIPOR1* (wk 9) in LL chickens, which suggests that adipose tissue of FL chickens could be less sensitive to insulin at these ages. Attractin (*ATRN*) is a neuropeptide involved in melanocortin signaling and regulation of food intake, which suppresses diet-induced obesity [66]. Our qRT-PCR analysis shows that the expression of *ATRN* markedly increases in abdominal fat after 5 wk of age in both the FL and LL; furthermore, the expression pattern of *ATRN* is strikingly similar to that of *ADIPOQ* and *ADIPOR1*. The adipokine *ANGPTL4* was expressed higher in LL chickens at 1, 5, 7 and 11 wk of age, although this difference was not statistically significant by qRT-PCR analysis. Originally, ANGPTL4 was identified as a secreted “fasting-induced adipose factor (FIAF)” in the mouse that was sharply up regulated by fasting and a target gene of the transcription factor PPARA [67]. In fact, ANGPTL4 is a potent irreversible inhibitor of lipoprotein lipase (LPL) activity, which leads to hypertriglyceridemia [68]. Our qRT-PCR analysis shows a 3-fold increase in expression of LPL in the LL chicken at 7 wk. Of particular interest, ANGPTL4 promotes the cleavage of LPL, while the proteolytic cleavage of ANGPTL4 by proprotein convertase releases a more potent inhibitor of LPL activity—the N terminal domain [69]. Thus, abdominal fat of chickens is enriched with adipokines, which can exert either local (autocrine/paracrine) or systemic (endocrine) actions after proteolytic processing and secretion into circulation (Table 3).

Our initial survey of global gene expression in abdominal fat of juvenile FL and LL chickens highlights another important feature of the avian endocrine system—the virtual absence of several important adipokines normally found in mammals. A few examples of adipokines not yet mapped to the current draft of the chicken genome (galGAL4), include leptin (*LEP*), omentin (*ITLN1)*, resistin (*RETN)*, tumor necrosis factor alpha (*TNFA*), and *PAI-1*. The existence of the chicken *LEP* gene remains a great and unresolved controversy [70-74], especially since extensive expressed sequence tag (EST) [75] and whole genome sequencing projects have failed to identify a *bona fide LEP* gene in the chicken. Furthermore, the *LEP* gene is absent from the genome of all birds sequenced so far (i.e., chicken, turkey, zebra finch, budgerigar and duck). However, the leptin receptor (*LEPR*) gene is expressed in several chicken tissues [75-79]; and chicken LEPR is capable of activating the JAK-STAT pathway *in vitro*[80,81]. Similarly, components of TNF signaling are up regulated in the hypothalamus of LL chickens [79], although *TNFA* is yet to be identified in chickens*.* Despite the absence of several mammalian adipokines (i.e., *LEP, TNFA, RETN, PAI-1*, *APOE*, and *ITLN1*) and metabolic enzymes (i.e., *LIPE*), adipogenesis and lipid metabolism in the chicken are robustly regulated by mechanisms that are, for the most part, similar to those described in mammals.

### Retinol metabolism and retinoic acid signaling in adipose tissue

Another remarkable observation from the present study was the over expression of 13 genes in abdominal fat of LL chickens that control metabolism of retinol, the precursor of retinoic acid (RA), which itself is a major chemical activator of multiple transcription factors controlling lipogenesis. The primary source of retinol is dietary plant-based β-carotene, which is symmetrically cleaved by the enzyme β-carotene monooxygenase 1 (BCMO1) into two molecules of retinal. Recently, we discovered mutations in the proximal promoter of *BCMO1*, which are responsible for variation in the color of breast meat in another F2 resource population of meat-type chickens [82]. Another enzyme, β-carotene oxygenase 2 (BCO2), asymmetrically cleaves one molecule of β-carotene to generate one molecule of retinal and a by-product (e.g., β-apo-14′-carotenal), which acts downstream to block signaling of PPARG [83]. The *BCO2* gene in chickens was originally identified as the *yellow skin* gene, which controls the β-carotene content and thereby yellow pigmentation of the skin [84]. Our qRT-PCR analysis of these two β-carotene degrading enzymes (Figure 8), showed only a main effect of age on expression of *BCMO1*, whereas the abundance of *BCO2* was greater in abdominal fat of LL chickens, producing a main effect of genotype. Another study found increased expression of *BCO2* in adipocytes from *BCMO1* knockout mice and that dietary β-carotene reduces adiposity of mice—but only in the presence of a functional BCMO1 enzyme [85]. This study also demonstrates the importance of BCMO1 in generating the precursor (retinal) for RA, which inhibits activation of PPARG and its lipogenic target genes that are mainly metabolic enzymes, adipokines and transport proteins (see Additional file 6). Our study shows higher expression of both *BCMO1* and *BCO2* in abdominal fat of the LL chickens after 5 wk of age, which presumably would lead to generation of more retinal and RA. This idea is supported by the differential expression of several genes involved in retinol metabolism and RA signaling in adipose tissue of FL and LL chickens. These genes are involved in transport of retinol (*RBP4, TTR* and *RBP7*), metabolism of retinol (*RDH1*, *RETSAT*, *ADH1C, ADH5,* and *CYP2E1*), and respond to RA (*RARRES2*, *GPRC5C*, and *NOL7*). In 3T3-L1 preadipocytes, RA inhibits adipogenesis via up-regulation of the transcriptional modulator SMAD3 [86]. Interestingly, two members of the SMAD family (*SMAD5* and *SMAD6*) were up-regulated (main effect of age) in adipose tissue of LL chickens (Additional file 3). The ligand (RA) activates its nuclear receptors (RAR and RXR), which can form heterodimers with other ligand-dependent transcription factors (e.g., LXR, PPARG and THR) to initiate transcription of numerous downstream target genes. Thus, RA seems to play an important role in reduction of adipogenesis and adiposity in the LL chickens.

### Visceral adipose tissue as a major site of lipogenesis in chickens

Because the liver is widely considered as the primary site of *de novo* lipid synthesis in birds, most transcriptional studies of lipogenesis in the chicken have focused on liver rather than adipose tissue. A targeted low-density array enabled an initial transcriptional analysis of liver [at a single age (8 wk)] in the FL and LL chickens [32]. This study showed up-regulation of several lipogenic genes (*ACACA*, *FASN*, *SCD*, *APOA1*, *SREBF1*, and *MDH2*) in the FL chickens. Examination of hepatic gene expression at three ages (1, 4, and 7 wk) in another population of chickens divergently selected on abdominal fatness revealed differential expression of several genes involved in lipid metabolism, including *ACAT1*, *CEBPγ*, *FABP1*, *APOA1*, *MDH1*, *APOD* and *PPARG*[87]. A time-course (1–11 wk) transcriptional study of liver in juvenile FL and LL chickens revealed 1,805 differentially expressed (DE) genes, mostly between 7 and 11 wk [3]. These functional genes identified in the liver of juvenile FL and LL birds were transcription factors, metabolic enzymes, transport proteins, differentiation factors, signaling molecules and adipokines.

In contrast, there have been only a few transcriptional studies of adipose tissue in the chicken. For example, a comparison of abdominal fat between meat-type (broiler) and egg-type chickens (layer) at a single physical age (10 wk), albeit at different physiological ages, focused attention on the up-regulation of *LPL* in broiler chickens and higher expression of *APOA1* in layers [88]. Another study using abdominal fat samples taken at 7 wk from a different population of divergently selected fat and lean chickens reported the differentially expression of 230 adipose genes [153 were up-regulated in the fat chickens, while 77 were up-regulated in the lean birds] [89]. Their conclusion that *TNFA* plays a key role in lipid metabolism of the chicken is surprising, since this adipokine has not been mapped to the chicken genome sequence. A recent transcriptional study of chicken abdominal fat compared a short-term (5 hr) fasting response with acute insulin immunoneutralization [21]. Surprisingly, fasting provoked larger changes in adipose gene transcription (1,780 DE genes) than did insulin neutralization with only 92 DE genes, which confirms the insensitivity of chicken abdominal fat to insulin [22]. In contrast, more than a thousand genes were either differentially expressed in liver or leg muscle of the same birds following insulin immunoneutralization [20]. Nonetheless, short-term fasting in the chicken depressed the expression of 40 genes in abdominal fat that are involved in the synthesis and storage of lipid, while a number of adipose genes that control lipolysis and oxidation of fatty acids were up-regulated by fasting or insulin neutralization [21].

The present study has identified a large number of lipogenic genes that are up regulated in abdominal fat of FL chickens (Table 3). A prime example of this lipogenic group is our clone for GH1 (GenBank accession BI390457) that corresponds to the short form of chicken GH (scGH), which lacks a signal peptide and is highly expressed in ocular tissue [90,91], pituitary gland and heart of chick embryos [91]. The short alternatively-spliced (16.5 kDa) isoform of full length GH (20 kDa) appears to function as an “intracrine” factor within the cell [91]. Our discovery of higher expression of scGH in abdominal fat of the FL chicken supports the idea of a local lipogenic action of GH on adipose tissue, rather than the lipolytic response usually observed in mammals [92]. In fact, our earlier work clearly established the lipogenic action of exogenous GH in juvenile chickens [93-96].

Up-regulation of two transcription factors (*SREBF1* and *THRSPA*) and the histone deacetylase *SIRT2* in abdominal fat of the FL were accompanied by higher expression of multiple genes involved in the generation and metabolism of lipids (*DHCR7, FADS2, FASN, HMGCR, HMGCS2, LSS, MVD, SCD* and *SC5DL*). The higher expression of the transcription factor *SREBF1* and 12 lipogenic target genes in the FL strongly supports our idea that the divergence in abdominal fatness of FL and LL chickens could be related to differential expression of several lipogenic genes in abdominal fat of the FL. For example, *FADS2*, which catalyzes the rate limiting step in synthesis of highly unsaturated fatty acids, was highly up regulated in abdominal fat of FL chickens; binding sites for both SREBF1 and PPARA are found in the promoter region of *FADS2*[97]. Likewise, SREBF1 regulates transcription of several genes that control synthesis of fatty acids, including acetyl-CoA carboxylase alpha (*ACACA*), which catalyzes the rate-limiting step in fatty acid synthesis [98,99]. Furthermore, MID1 interacting protein 1 (MID1IP1) [or THRSP-like (THRSPL)] enhances ACACA polymerization and its enzymatic activity [100]. Adipose tissue from FL chickens shows higher expression of *THRSPA*, a transcriptional regulator of several lipogenic genes in the chicken [3,78,96,101]. Earlier, we discovered a 9-base pair deletion near the putative DNA-binding domain of chicken *THRSPA* and demonstrated association of this insertion/deletion polymorphism with abdominal fatness traits in multiple resource populations of chickens [101]. Mutations in the *THRSPA* gene of chickens [101-103], ducks [104] and geese [105] are associated with fatness traits and are perhaps of potential use as molecular markers in poultry breeding programs. Furthermore, THRSP is a major regulator of adipogenesis in skeletal muscle of beef cattle [106,107] and of lipogenesis in the lactating mammary gland of the dairy cow [108-110]. Interestingly, the THRSP-null mouse shows reduced lipogenesis in the mammary gland [111] and pups from the THRSP-null mouse exhibit reduced body weight gain due to diminished milk triglycerides [112]. In humans, amplification of the THRSP locus is associated with lipogenic breast cancer [113]; and, as such, THRSP serves as a marker of aggressive breast cancer and a potential target of anti-cancer drugs [114]. In humans, expression of *THRSP* in adipose tissue is depressed by transition from a lipogenic fed state to a lipolytic state induced by a 48 hr fast [115]. These observations support the idea that THRSP is a transcriptional activator of several lipogenic enzymes (ACLY, FASN and ME) in the mouse [116]. THRSP is activated in response to T_3_, glucose and insulin and inhibited by polyunsaturated fatty acids [117], cyclic AMP or glucagon [118]. Recent work has shown that induction of THRSP increases expression of FASN in cultured hepatocyte cells and RNAi-mediated knock-down of THRSP depresses expression of FASN [119]. Another study showed that FASN co-precipitates with THRSP in nuclear extracts from the mouse (referenced in [110]). The exact mechanism by which THRSP and MID1IP1 interact and work as regulators of gene transcription is currently unknown. These genes are highly expressed in fatty tissues of birds and mammals, where they regulate the expression and activity of multiple lipogenic enzymes. The proximal (4 kb) promoter region of *THRSPA* contains four putative binding sites for PPARG and four SREBF sites (L.A. Cogburn, unpublished observations). In the present study, we found higher expression of *THRSPA* in abdominal fat of FL chickens at all ages, except at 7 wk. In the rat, the far-upstream region of the *THRSP* promoter contains three T_3_-THR response elements (TREs) [120]. Thus, *THRSPA* is responsive to metabolically active thyroid hormone (T_3_) generated by the activation enzyme *DIO1*, whereas the enzyme DIO3 is responsible for degradation of metabolically active T_3_ and conversion of the prohormone (T_4_) to metabolically inactive reverse T_3_ (rT_3_) [121]. The up regulation of *DIO3* in adipose tissue of juvenile LL chickens (1–11 wk) suggests that less T_3_ would be available to activate *THRSPA* transcription, which was observed in the LL. Thioredoxin interacting protein (TXNIP) is another important regulator of hepatic glucose metabolism [122] that also mediates hypothalamic control over energy utilization and adiposity in the mouse [123]. The up-regulation of *TXNIP* in abdominal fat of the FL during the period of maximal fatness (3–11 wk) could contribute to their enhanced lipogenesis and adiposity. Likewise, we have discovered another putative sensor of glucose, the sweet taste receptor 1 (*TAS1R1*) gene, which is differentially expressed in the hypothalamus [79] and abdominal fat (Figure 2) of FL and LL chickens. Our observation of higher expression of *TAS1R1* in the hypothalamus of the FL and abdominal fat of the LL suggest tissue specific regulation of this important tissue glucose sensor [124-126].

### Increased lipolysis in abdominal fat of LL chickens

In contrast to the enhanced lipogenic state found in abdominal fat of FL chickens, the LL show higher expression of numerous genes involved in lipolysis (Table 3). Two cytochrome P450 family members (*CYP27A1* and *CYP2E1*) were expressed at higher levels in abdominal fat of the LL when compared to FL chickens. *CYP27A1* is involved in clearance of cholesterol via bile excretion, whereas *CYP2E1* is strongly induced in white adipose tissue of the rat by prolonged fasting [127]. The beta-subunit (*HADHB*) of mitochondrial tri-functional protein (MTP), a complex that catalyzes the final three steps of β-oxidation of long chain fatty acids, was also up regulated in adipose tissue of LL chickens. MTP knockout mice exhibit neonatal hypoglycemia and sudden neonatal death indicating its essential role in β-oxidation of long chain fatty acids [128]. Two members of the hydroxysteroid (17-β) dehydrogenase family (17β-HSD, members 4 and 6) were also expressed higher in adipose tissue of the LL. The significance of HSD17B4 in β-oxidation of branched chain fatty acids was demonstrated in HSD17B4 knockout mice, which were unable to degrade phytanic and pristanic acids [129]. Since the other 17β-HSD (HSD17B6) exhibits retinol dehydrogenase activity [130], its up-regulation in adipose tissue of the LL chicken suggests increased availability of all trans-retinoic acid. In addition, *PDK4*, which inhibits the pyruvate dehydrogenase complex and conversion of pyruvate to acetyl-CoA [131], was highly up-regulated in abdominal fat of the LL between 7 and 11 wk. The high expression of *PDK4* in the liver of chicken embryos [3], whose energy is derived exclusively from yolk lipids, supports a similar action of PDK4 in adipose tissue of the LL chickens. Furthermore, the expression of *PDK4* increased sharply in abdominal fat of two-week-old chickens by a 5-h fast or insulin immunoneutralization [21]. The tumor suppressor protein p53 enhances lipid catabolism and induces expression of guanidinoacetate N-methyltransferase (GAMT), which enhances β-oxidation of fatty acids [132]; both of these genes were up regulated in visceral fat of the LL chickens. Another gene up regulated in the LL that protects against oxidative stress is *SOD3*, which is expressed at higher levels in the liver of low-growth (leaner) chickens [3]. Thus, the present time-course transcriptional analysis of abdominal fat in juvenile FL and LL chickens provides compelling evidence for enhanced lipolysis in adipose tissue of the LL.

## Conclusions

The present study adds a new dimension to function of visceral fat as a proteolytic processor of adipokines and other endocrine signals that control lipid metabolism. In particular, the LL cockerels exhibit high expression of several blood coagulation factors in adipose tissue, albeit not in liver. Some of these changes in the LL occur before the divergence in fatness. These hemostatic proteases and protease inhibitors could be involved in activation of adipokines, chemokines and other metabolic ligands that contribute to suppression of lipogenesis and adipogenesis in the LL. Furthermore, abdominal fat of the LL chickens has higher expression of genes involved in mobilization, utilization and export of lipids than does the FL. Several transcription factors have a larger number of target genes expressed higher in the LL that could also favor suppression of abdominal fat accretion. In contrast, abdominal fat of the FL chickens expresses a greater abundance of numerous target genes involved in lipogenesis and adipogenesis, which could contribute to their greater adiposity. The higher expression of these target genes in FL chickens appears after the onset of divergence in fatness. Therefore, abdominal (visceral) fat of the chicken could play a more significant role in lipogenesis and adiposity than previously considered. The assumption that the liver of birds serves as the major site of lipogenesis needs to be re-examined.

### Availability of supporting data

The minimum information about microarray experiments (MIAME)-compliant microarray data described in this article are available in the NCBI Gene Expression Omnibus (GEO) under the accession number GSE37585. Additional file 3 provides annotated DE gene sets from statistical analysis of the microarray data; whereas Additional files 4, 5, 6 provide annotation, functional analysis and mapping of DE genes to biological functions and canonical pathways by IPA software.

## Abbreviations

FL: Fat line chickens; LL: Lean line chickens; DE: Differentially expressed; G: Main effect of genotype; A × G: Age by genotype interaction; A: Main effect of age; wk: Week of age; eQTL: Expression quantitative trait loci; GGA5: *Gallus gallus* chromosome 5; IPA: Ingenuity Pathway Analysis; INRA: Institut National de la Recherche Agronomique; qRT-PCR: Quantitative reverse transcriptase polymerase chain reaction; h: Hour(s); L: Light; D: Dark; LIMMA: Linear models for statistical analysis of microarray data; FDR: False discovery rate; SAS: Statistical Analysis System; GLM: General linear models; LSMEANS: Least Squares Means.

## Competing interests

The authors declare that they have no competing interests.

## Authors’ contributions

LAC, SEA, JS, ELBD, MJD and TP secured funding, designed and carried out the animal experiment. LAC, WC, XW and TEP designed, constructed and printed the Del-Mar 14 K Chicken Integrated Systems microarrays. WC isolated RNA from the 48 abdominal fat samples, labeled, hybridized and scanned the microarrays. CWR performed statistical analysis of microarray data, completed the Ingenuity Pathway Analysis (IPA) and qRT-PCR analyses, and wrote the first draft of the manuscript. All coauthors reviewed, revised, commented on and approved the final version of the manuscript.

## Supplementary Material

Additional file 1 **Microarray experimental design.** A Microsoft Excel file containing a single work sheet “Array Hybridization Design” describes the balanced block hybridization scheme used for the time-course microarray analysis of abdominal fat in FL-LL chickens. Click here for file

Additional file 2 **Description of the primers used for the qRT-PCR analysis.** A Microsoft Excel file containing a single work sheet “Primer information” provides the gene symbol, gene name, forward and reverse primer sequences, GenBank accession and amplicon size (bp) for each primer used for qRT-PCR analysis. Click here for file

Additional file 3 **Differentially expressed (DE) gene lists.** A Microsoft Excel file containing three work sheets (“Main Effect of Genotype”, “Age X Genotype Interaction” and “Main Effect of Age”) that provide information about DE genes identified as the main effect of genotype, age x genotype interaction, or the main effect of age, respectively. Each list provides the clone ID, gene symbol, gene name, Entrez protein ID, log2 ratio (FL/LL) and FDR adjusted *P*-value for each DE gene. Click here for file

Additional file 4 **IPA summary of DE genes related to diseases and disorders.** A Microsoft Excel file containing six work sheets. “Overall IPA Summary” presents a synopsis of functionally annotated diseases and disorders in abdominal fat of FL-LL chickens. This work sheet provides information on IPA functional annotation, *P*-value for over-representation of DE genes, a list of genes and the number of genes assigned to each biological function. Five worksheets provide annotation of DE genes listed in “Overall IPA Summary”. Each worksheet provides the Entrez protein ID, gene symbol and log2 ratio for functionally annotated groups of genes. Click here for file

Additional file 5 **IPA canonical pathways in abdominal fat of FL and LL chickens.** A Microsoft Excel file containing a single worksheet “Canonical Pathways”. The top of the worksheet provides the canonical pathway, *P*-value associated with over-representation and ratio (number of genes present in dataset/number of known genes assigned to that pathway in the Ingenuity® Knowledge Base. Below the summary, a table for each canonical pathway provides the gene symbol and log2 expression ratio (FL/LL). Click here for file

Additional file 6 **IPA annotated DE genes in their molecular and cellular function.** This Microsoft Excel file contains two worksheets: “IPA Summary” presents nine functional categories of DE genes found in abdominal fat of the FL and LL chickens that are related to “Lipid Metabolism”. This worksheet provides the biological function, *P*-values of over-representation, the number of genes, and a gene list for each functional category. Four of the functionally annotated groups (bold type) are expanded in the second worksheet “Lipid Metabolism” to include protein ID, gene symbol, and log2 expression ratio (FL/LL). Click here for file

Additional file 7 **Verification of differential expression of transcription factors by qRT-PCR analysis.** This PowerPoint slide file shows qRT-PCR analysis of six transcription factors. Each data point represents LSMEANS (n = 4 birds/genotype) of normalized expression values. A two-factor ANOVA was used to determine significance (*P*≤0.05). The shaded box in each panel indicates significant effects of age (A), genotype (G) and/or the A x G interaction; the parenthesis shows the common standard error (SE) of LSMEANS for that gene as determined by the GLM procedure in SAS. Click here for file

Additional file 8 **qRT-PCR analysis of genes involved in thyroid hormone and retinol metabolism and signaling.** The abundance of genes involved in signaling and metabolism of thyroid hormone (A., left side) and retinol (B., right side) was verified by quantitative reverse transcription PCR (qRT-PCR) analysis. Each data point represents LSMEANS (n = 4 birds/genotype) of normalized expression values. A two-factor ANOVA was used to determine significance (*P*≤0.05). The shaded box in each panel indicates significant effects of age (A), genotype (G) and/or the A x G interaction; the parenthesis shows the common standard error (SE) of LSMEANS for that gene as determined by the GLM procedure in SAS. Click here for file
