# Supplementary figures and images for: Transcriptional analysis of abdominal fat in genetically fat and lean chickens reveals adipokines, lipogenic genes and a link between hemostasis and leanness
Source: BMC Genomics. 2013 Aug 16;14:557. doi: 10.1186/1471-2164-14-557 (PMC3765218; doi:10.1186/1471-2164-14-557)

## Slide 1
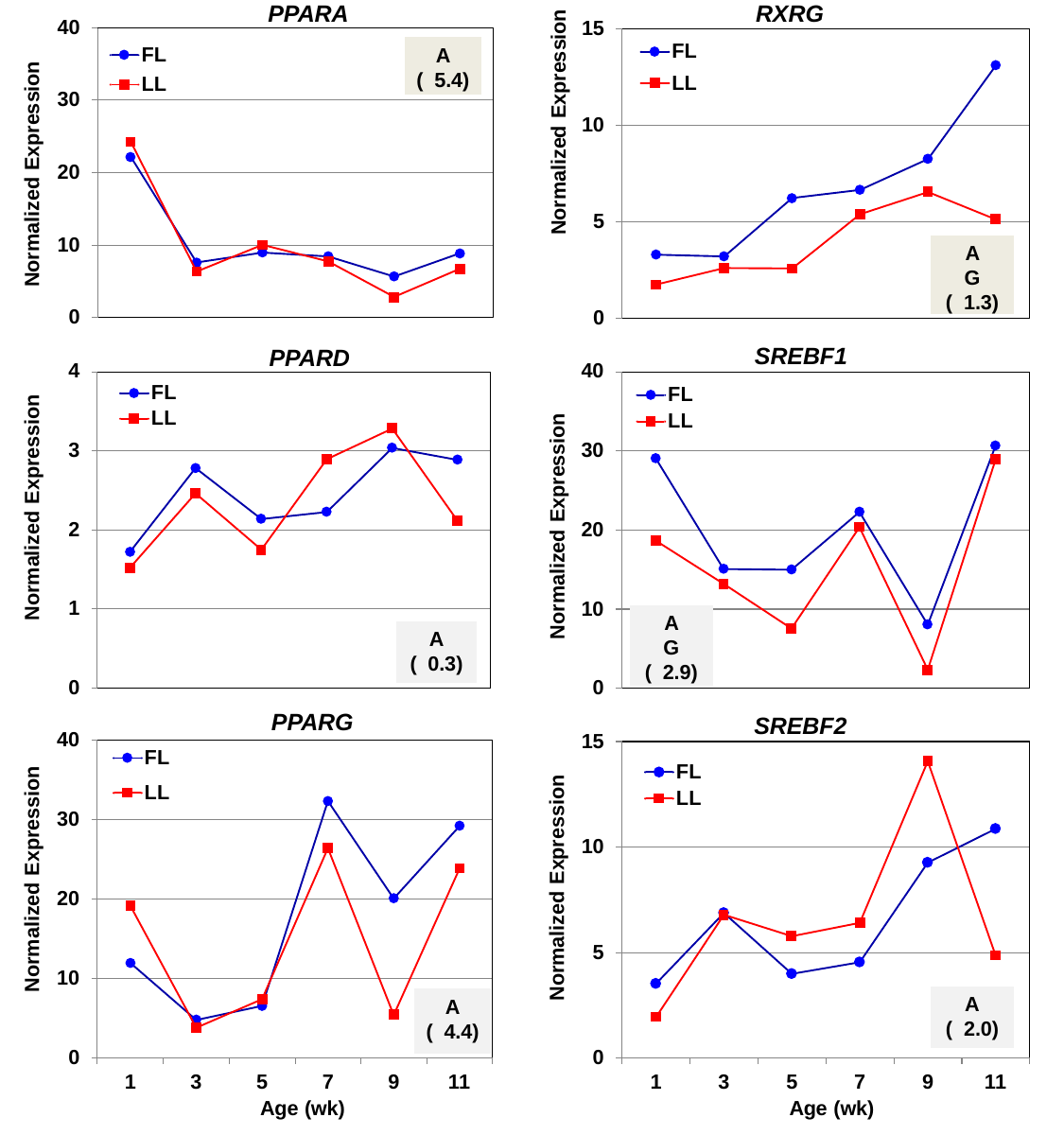

Supplement: Additional file 7 — Verification of differential expression of transcription factors by qRT-PCR analysis. This PowerPoint slide file shows qRT-PCR analysis of six transcription factors. Each data point represents LSMEANS (n = 4 birds/genotype) of normalized expression values. A two-factor ANOVA was used to determine significance (P≤0.05). The shaded box in each panel indicates significant effects of age (A), genotype (G) and/or the A x G interaction; the parenthesis shows the common standard error (SE) of LSMEANS for that gene as determined by the GLM procedure in SAS. [file 1471-2164-14-557-S7.pptx]

## Slide 1
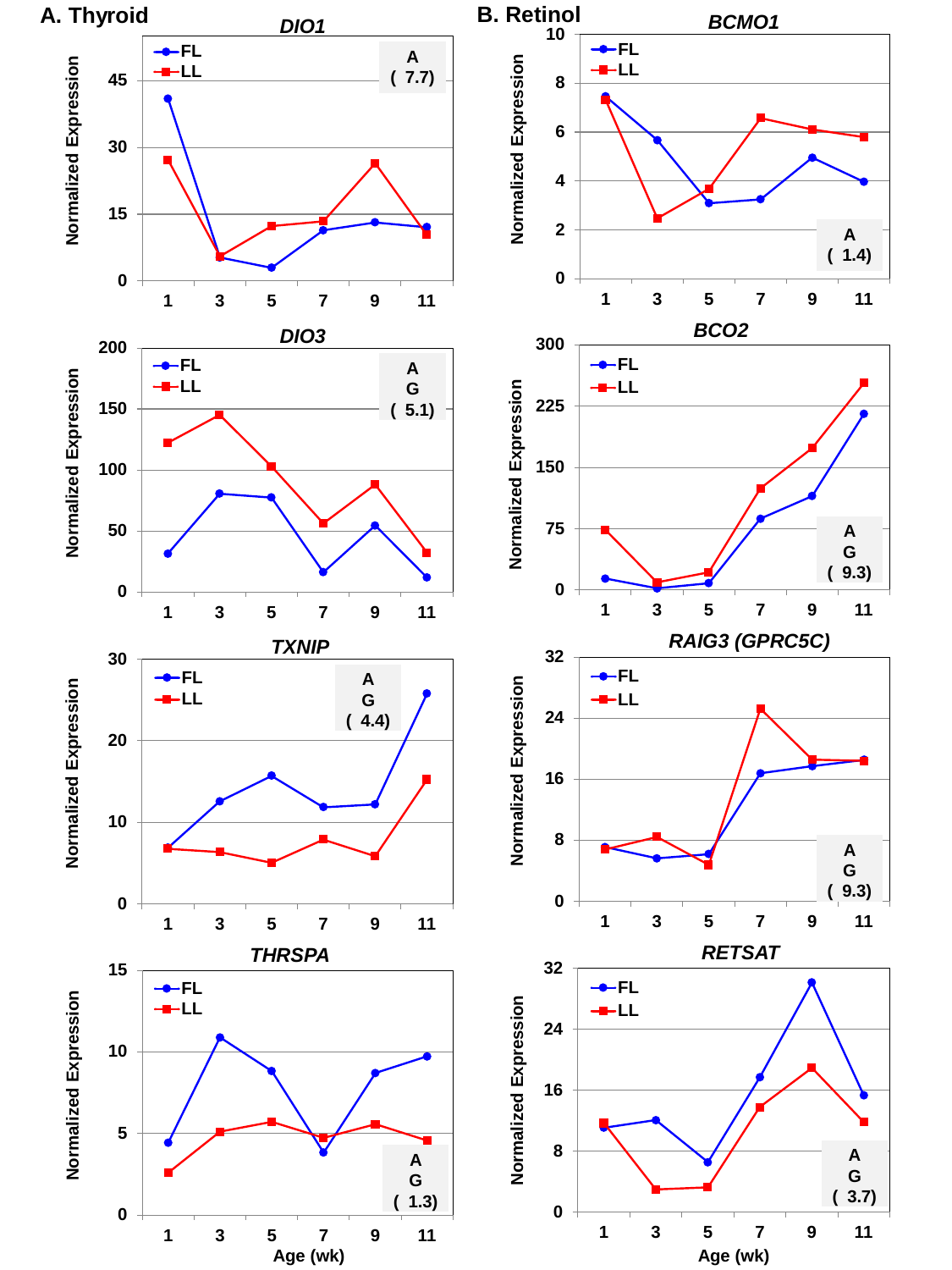

Supplement: Additional file 8 — qRT-PCR analysis of genes involved in thyroid hormone and retinol metabolism and signaling. The abundance of genes involved in signaling and metabolism of thyroid hormone (A., left side) and retinol (B., right side) was verified by quantitative reverse transcription PCR (qRT-PCR) analysis. Each data point represents LSMEANS (n = 4 birds/genotype) of normalized expression values. A two-factor ANOVA was used to determine significance (P≤0.05). The shaded box in each panel indicates significant effects of age (A), genotype (G) and/or the A x G interaction; the parenthesis shows the common standard error (SE) of LSMEANS for that gene as determined by the GLM procedure in SAS. [file 1471-2164-14-557-S8.pptx]
